# Social manipulations trigger shifts in group-level dominance patterns

**DOI:** 10.1371/journal.pcbi.1014875

**Published:** 2026-11-05

**Authors:** Annemarie van der Marel, Xavier Francis, Claire L. O’Connell, Cesar O. Estien, Chelsea Carminito, V. Darby Moore, Elizabeth A. Hobson

**Affiliations:** 1 Department of Biological Sciences, University of Cincinnati, Cincinnati, Ohio, United States of America; 2 Department of Biological Sciences, Thomas More University, Crestview Hills, Kentucky, United States of America; 3 Department of Environmental Science, Policy, and Management, University of California–Berkeley, Berkeley, California, United States of America; 4 Second Nature Ecology & Design LLC, Berkeley, California, United States of America; University of California Irvine, UNITED STATES OF AMERICA

## Abstract

Within dominance hierarchies, individuals often direct aggression based on their own rank and the rank of others. When many group members make the same rank-informed aggression decisions, a broader group-level “dominance pattern” can emerge which further structures aggression within the framework of the dominance hierarchy. However, whether groups can change their group-level dominance patterns over time, and what factors might drive these changes, are not well understood. We investigated aggression dynamics by experimentally manipulating captive groups of monk parakeets (*Myiopsitta monachus*) using targeted rank-based removals and reintroductions of group members to induce social instability. Dominance patterns often shifted after experimental perturbations but remained stable in their absence. The rank of the perturbed individual affected both individual aggression decisions and the shifts in dominance patterns observed. In particular, reintroductions of top-ranked birds led to marked changes in aggression and shifts in dominance patterns. Understanding the factors that shape these dynamics can help explain how animal societies respond to social disturbance.

## Author summary

Animals in social groups interact with each other and these interactions shape their social structure. Many species are structured by rank and dominance hierarchies. Rather than interacting randomly, animals may choose their fights based on information about each other’s ranks, using dominance patterns. For example, animals may choose fights with similarly-ranked individuals or with individuals ranked much lower than themselves. While these patterns occur across many animal species, it is not clear whether they can change over time within a group. To test this, we removed and reintroduced individual monk parakeets of different ranks in captive groups. We found that dominance patterns did shift and that changes were affected by rank: the reintroduction of top-ranked birds resulted in groups changing their dominance pattern to “bullying” where they switched to fighting individuals ranked far below themselves. Our results showed that parakeet social behavior is flexible and can respond to perturbations and that rank is an important factor in these changes. Understanding the factors that drives these dynamics can help identify when social structures are stable or more likely to reorganize.

## Introduction

Social systems are inherently dynamic: individuals join or leave groups, relationships between individuals form or weaken, and social networks rewire [1–5]. In dynamic social systems, individuals must adjust their behavior accordingly [6]. The ability to respond to changes in social environments, or social plasticity, is likely crucial for navigating complex social systems, such as in groups with *dominance hierarchies*, where *aggression* shapes social relationships and rank structure [7,8], (terms defined in Table 1).

**Table 1:** Glossary.

Term	Definition
Aggression	Behavior that threatens, displaces, injures, or imposes other costs on another individual
Aggression balance	The aggression given divided by the total aggression (sum of aggression given and received by each individual)
Aggression dynamics	Changes in the frequency, direction, or intensity of aggressive interactions over time
Dominance	Asymmetry in aggression by one animal towards another animal [20–22]
Dominance hierarchy	Group-level social structure shaped by relatively stable, asymmetric dominance relationships among group members. These relationships are established interactions, such as aggression, displays of threat, avoidance, or submission, in which some individuals consistently are the actors and others the receivers, allowing individuals to be ranked relative to others
Dominance rank	An individual’s position within a dominance hierarchy relative to others
Dominance pattern	An emergent group-level pattern that occurs within the structure of the group’s dominance hierarchy where group members make similar rank-informed aggression decisions: *downward heuristic* (aggress towards any ranked lower than self), *close competitors* (preferentially attack those ranked slightly lower than self), and *bullying* (preferentially attack those ranked much lower than self) [7]
Perturbation	The experimental procedure where we removed or reintroduced a single focal individual (an individual chosen based on its dominance rank); both removals and reintroductions were treated as “perturbations”
Rule-following and -breaking	Rule-followers are higher-ranking individuals that aggress against lower-ranking individuals. Rule-breakers are lower-ranking individuals that aggress against higher-ranking individuals

Within a group’s dominance hierarchy, an individual’s decisions about how to direct aggression can be based on their own rank and the rank of potential opponents. When multiple individuals make similar rank-informed aggression decisions, a group-level pattern can emerge, that further structures aggression patterns within the confines of the dominance hierarchy [7]. Consistent with [7], we refer to these group-level aggression patterns as *social dominance patterns* (Table 1). These dominance patterns describe the categorization of aggression across a group, rather than a property of a dominance hierarchy, such as steepness, or linearity.

Across animal social systems, emergent group-level patterns are well-documented, where simple individual rules result in collective behavior and give rise to structured behavior at the group-level, such as collective movement or synchronized vigilance [9–11]. Within the context of aggression and hierarchies, these dominance patterns are emergent properties that arise from the consensus of individual aggression decisions across the group, not traits of single individuals. This distinction between individual behavior and group-level emergent patterns is central to understanding social plasticity: while individuals adjust their aggression, the outcome can be a reorganization of the group’s overall social structure.

Dominance patterns are identified by comparing two parameters describing the distribution of aggression—its concentration on receivers at similar versus different rank distances and its position within the hierarchy—with values simulated from reference models [7]. Because this classification is based on each group’s observed values relative to its own reference distribution, similar parameter values may receive different classifications in distinct groups. Recent work identified three dominance patterns: 1) *downward heuristic* (aggress towards any ranked lower than self), 2) *close competitors* (preferentially attack those ranked slightly lower than self), and 3) *bullying* (preferentially attack those ranked much lower than self) [7] (Table 1). The downward heuristic can be thought of as a null model for aggression within a hierarchy, as there is no additional structuring of aggression beyond the patterns expected if individuals are randomly choosing to fight any individual ranked below themselves. Both close competitors and bullying respect these basic “rules” of aggression within a hierarchy, but represent additional structuring that occurs within the confines of the hierarchy.

Previous work documented dominance patterns across a large range of species and found no evidence that any of these patterns were phylogenetically restricted [7]. Importantly, this work also showed that dominance patterns should not strictly be considered a species-level characteristic, because when multiple groups were sampled within species, different groups could and did show variability in which dominance patterns they used [7]. However, this previous work could not determine whether these groups consistently used different dominance patterns, or could change the dominance patterns they used over time. Understanding the conditions under which groups might change dominance patterns would provide insight into the social plasticity of structured aggression and how changes in individual decision-making could result into group-level reorganization.

One condition that may induce social plasticity in aggression and dominance patterns is changes in group membership, i.e., membership instability [12], although such dynamics are relatively hard to follow under wild conditions. To test this possibility, we performed social perturbation experiments in which an individual was removed from a stable social group, the group was allowed to restabilize, and the removed individual was reintroduced after the re-stabilization period in captivity. Within four captive groups of monk parakeets (*Myiopsitta monachus*), we tested whether removals and reintroductions, i.e., a perturbation (Table 1), of focal individuals differing in *dominance rank* would 1) alter the aggression dynamics and 2) trigger dominance pattern shifts. Monk parakeets are well-suited for studying social dynamics as they are highly social parrots that use short-term social history in affiliative and aggressive decision-making [13–15], readily form dominance hierarchies in captivity [13,16–18], can develop and follow different dominance patterns [7,18], and exhibit high fission-fusion dynamics in both wild and captive populations [16]. Earlier work with the same social groups and perturbation experiments focused on individual changes in dominance rank and whether rank was determined by individual characteristics or short- or long-term social history, i.e., the memory of social interactions [13]. This study found that all focal birds (independent of rank) did not reoccupy their previous rank position upon reintroduction implying that short-term social history (the interactions during the removal period) was more important in determining rank compared to individual characteristics and longer-term social history [13]. In the current study, we used data from the same experiments, but we asked whether experimental perturbations of group membership would trigger changes in aggression dynamics that scale up to shifts in the group’s dominance pattern, i.e., the emergent, group-level structure that arises from those aggression dynamics.

First, we hypothesized that if aggression dynamics and dominance patterns are plastic, our perturbations would result in individuals redirecting their aggression toward different subjects and to alter the rate and distribution of their aggressive interactions. These individual-level changes in aggression would lead to shifts in the overall dominance pattern of the group compared to the pre-perturbation period.

Second, we hypothesized that dominance rank (top-ranked or lower-ranked) and perturbation type (removals and reintroductions) would differentially induce changes in aggression dynamics and dominance patterns. We predicted that top-ranked perturbations and removals compared to lower-ranked perturbations and reintroductions, respectively, would lead to higher aggression rates, greater *aggression balance* (aggression given/[aggression given + received], Table 1) differences, more shifts in dominance patterns, and different dominance pattern shifts. We predicted that the perturbation of top-ranked individuals would have a greater impact because we considered these to be ‘key’ individuals with significant impact on social dynamics [19]. We predicted that removals would have a greater impact than reintroductions because the sudden absence of a key individual could provide an opportunity for the remaining members to update their ranks. In contrast, after reintroduction, most of the group would have adjusted their ranks, except for the returning bird [13]. Because our previous work showed that reintroduced individuals did not regain their previous rank [13], we expected that the remaining birds would not change their aggression decisions and that dominance patterns would remain stable.

## Results

We experimented with 51 unique individual monk parakeets housed in four captive groups across three years of study (2020–2022), resulting in 1,461 hours and 3,530 person hours of behavioral observations and a total of 118,219 observed aggressive events (Tables 2, A-C in S1 Appendix). We binned these aggressive events into 3-day assessment periods. For each 3-day period, we used observations of directional aggression where there were clear winners and losers and determined each individual’s dominance rank using a network-based ranking algorithm based on PageRank that calculated a continuous rank score (power score) within the group [7,13,23]. We then quantified individual aggression decisions by visualizing aggression networks and calculating number of aggressive events and aggression balance. We modeled aggression count and aggression balance as a function of time, focal rank (top-ranked vs. lower-ranked), focal status (focal bird vs. remaining group members), including group size and observation hours as covariates to account for differences in encounter opportunities among groups. We finally categorized the dominance pattern of the group (i.e., the emergent group-level pattern in which group members make similar rank-informed aggression decisions, Table 1) using a rank-based reference model [7,23]. This model uses two continuous parameters to classify groups into one of three dominance patterns (downward heuristic, close competitors, or bullying [7]): focus (how concentrated aggression is across rank distances), and position (where aggression is focused relative to the aggressor’s rank). For each group, observed focus and position values are compared to a reference distribution generated by a downward-heuristic model with a tuning parameter that varies the proportion of random aggression; patterns are assigned based on whether the observed values fall within, above, or below the 95% confidence region of this model. To test whether groups differently shifted patterns after top-ranked or lower-ranked perturbations or after removals and reintroductions, we quantified the observed number and type of shifts across the three experimental social groups. We then compared these observed pattern transitions to a reference model where we randomized the patterns over 1000 iterations per group (see methods and Fig A in S1 Appendix).

**Table 2:** Aggression summary of the number of aggressive interactions by group.

Group	Total aggressive interactions	Hours observed	Group size	No. 3-day periods	Mean ± SE per period
2020	3,148	61.4	20	3	1049.3 ± 133.2
2021 non-experimental phase	18,858	220.9	22/20	12	1885.8 ± 156.2
2021 experimental phase	23,076	255.5	20	10	1923.0 ± 135.3
2022-1	42,280	458.5	11	26	1626.2 ± 123.3
2022-2	42,403	464.4	11	26	1630.9 ± 101.7

### Aggression dynamics and dominance pattern shifts

When we compared subsequent 3-day periods to the baseline period (the 3-days before the first removal), we found that the number of aggressive interactions remained stable over time across social groups after accounting for hours observed and group size (mean ± SD = 1685 ± 559 interactions, LR test: λ = 32.0, P = 0.16; Table 2; Fig 1). We found no evidence that group size was associated with aggression events; larger groups (1803 ± 527 interactions) showed similar amounts of aggression events compared to smaller groups (1629 ± 571 interactions; LR test: λ = 2.5, P = 0.12; Table 2; Fig 1).

**Fig 1:**
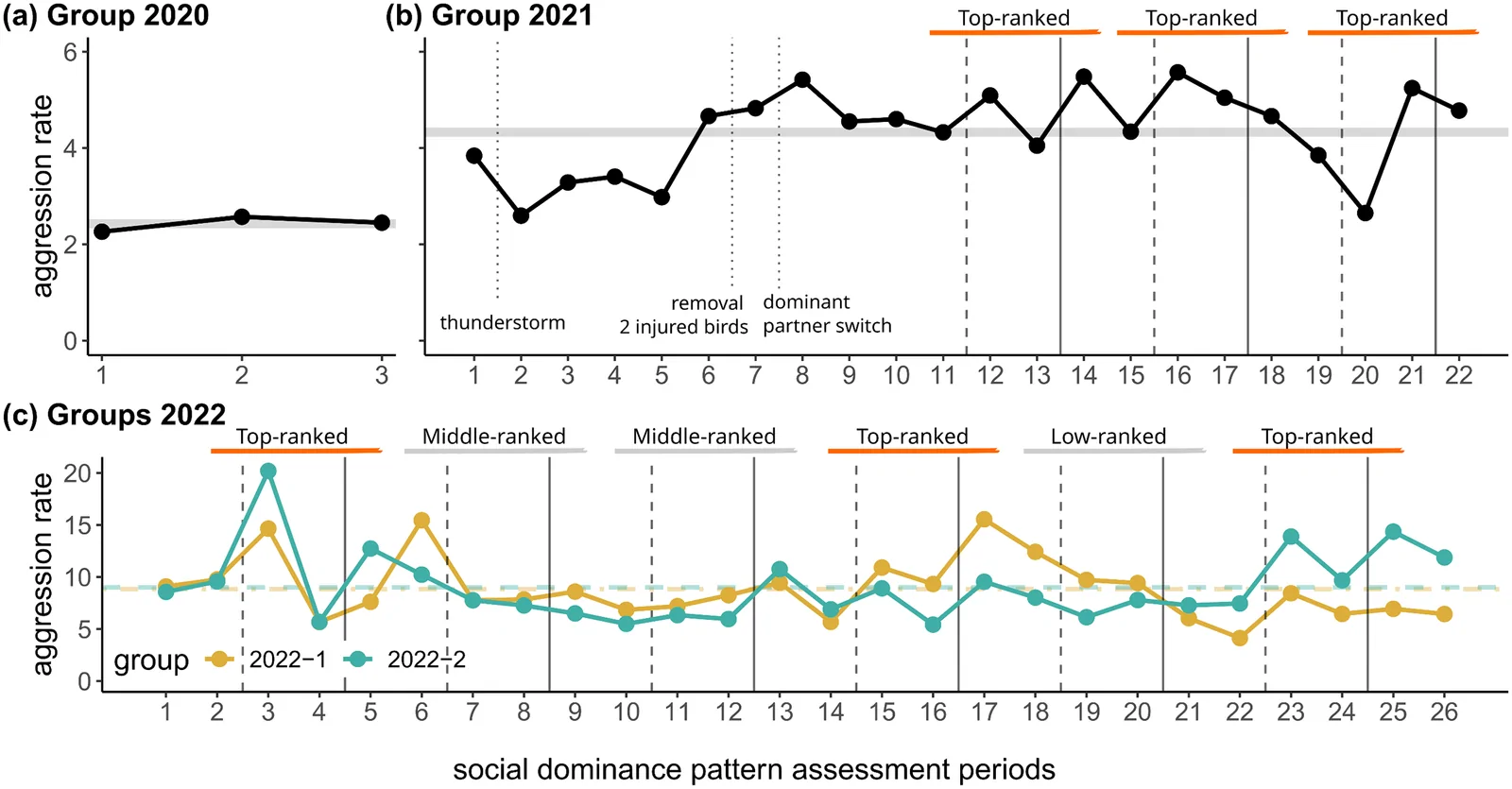
Aggression remained relatively stable for each social group. Each panel represents a different social group and shows the rate of directed aggression with the overall mean aggression rate (for panels (a) and (b) in grey, and for panel (c) horizontal dashed lines). Aggression was adjusted for group size and hours observed by standardizing counts to the number of potential interaction opportunities. Vertical lines indicate the timing of nonexperimental perturbations (dotted lines), and the experimental perturbations for removals (dashed lines) and reintroductions (solid lines). Note the different y-axis scale for panels a and b compared to panel c. The x-axes represent the 3-day binned assessment periods to quantify aggression.

We highlighted the change in the direction of aggression toward focal birds and remaining group members using aggression networks (Figs 2a, Fig B in S1 Appendix). The aggression networks showed that the identity of the target of aggression changed during our perturbation experiment (Fig 2a). Focal birds (i.e., single individuals that were removed and reintroduced based on their rank alone, and not their aggression balance) went from an aggression balance value closer to 1 (only aggress) before removal to a value closer to 0 (only receive aggression) upon reintroduction (Figs B and C in S1 Appendix).

**Fig 2:**
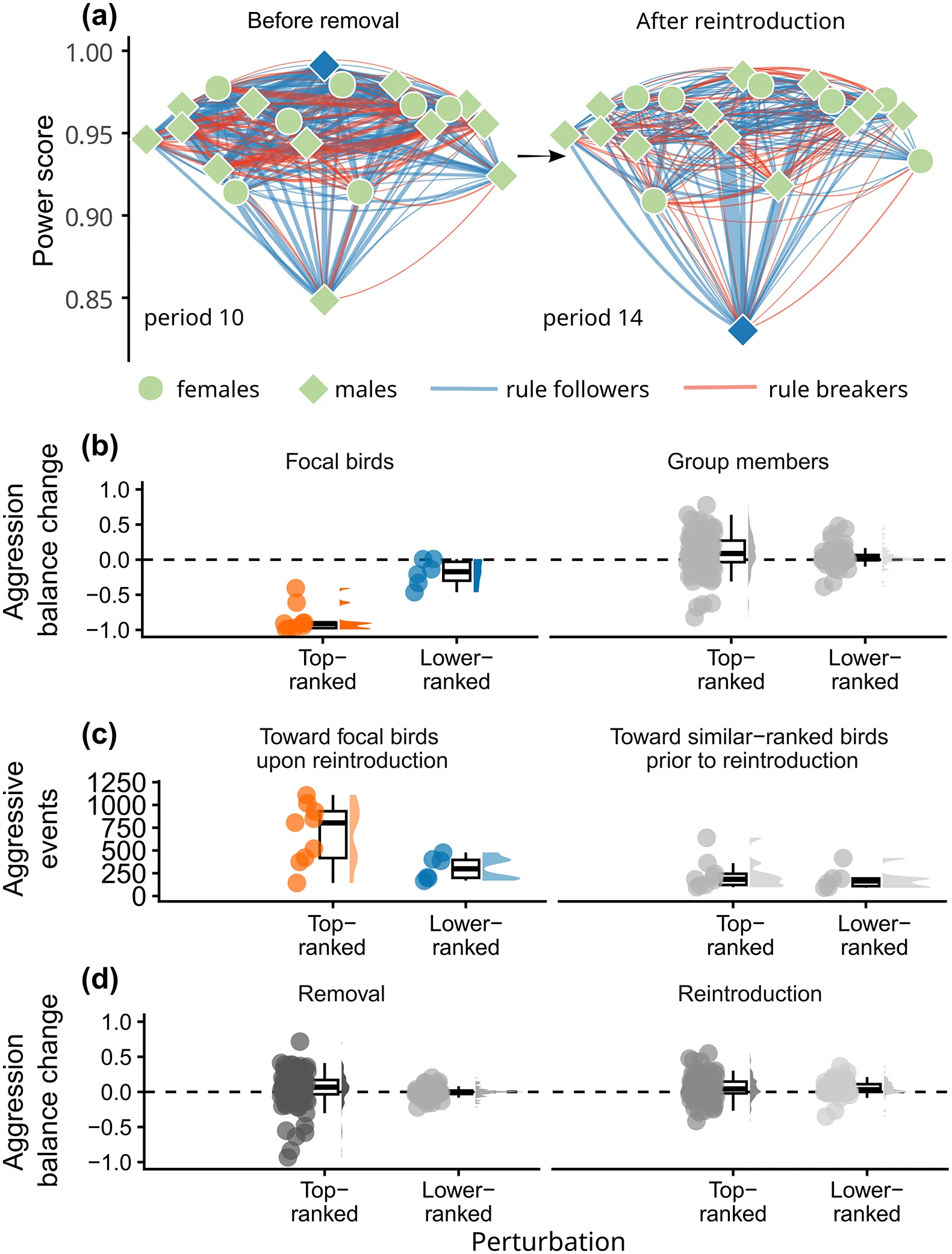
Aggression summary after perturbation experiments in captive monk parakeets. As example (a) shows the aggression networks for the first experimental perturbation in Group 2021 (the other perturbations are shown in Fig B in S1 Appendix). The nodes are ordered by their respective power score with higher ranking birds closer to 1. The focal bird is highlighted in blue. The edge width represents the total number of aggressive events between two individuals. The blue edges represent the rule-followers (higher-ranking birds aggress against lower-ranking birds), and the red edges represent the rule-breakers (lower-ranking birds aggress against higher-ranking birds). Panel (b) shows the change in aggression balance for the experimental groups, where we perturbed top-ranked or lower-ranked birds. We calculated the change in the aggression balance upon reintroduction minus the aggression balance before removal. Panel (c) reports the total number of aggressive events toward the focal birds during the assessment period upon reintroduction and toward the similar ranked bird in the assessment period prior to reintroduction of the focal bird. Panel (d) shows the change in aggression balance for the remaining group members before and after removal and before and after reintroduction for the top-ranked and lower-ranked perturbations. Top-ranked focal birds are colored in orange, lower-ranked focal birds are colored in blue, while the remaining group members are colored in different shades of grey dependent on the experimental perturbation.

Finally, we summarized the dominance patterns over time. Across all groups, we detected all three dominance patterns (Table 3, Figs 3, E, F, G in S1 Appendix). The most common pattern was the downward heuristic (attack any individual lower-ranked than self), which was found in 35 out of the 77 assessment periods (45%), followed by the bullying pattern (preferentially attack much lower-ranked individuals) in 25 out of 77 periods (32%), and the close competitors pattern (attack slightly lower-ranked individuals) in 17 out of 77 periods (22%; Table 3). When we did not experimentally perturb the social groups, we found that groups 2020 and 2021 non-experimental phase did not shift their dominance patterns during the observation period. The two groups differed in the dominance patterns they followed (Table 3): one consistently followed a bullying pattern (Fig 3a) and the other a downward heuristic pattern (Fig 3b until period 10).

**Table 3:** Summary of the dominance patterns across four social groups of captive monk parakeets. **We present the dominance pattern distribution as both count and percentage, and show the number of transitions after removals, reintroductions, perturbations, and during the stabilization period (i.e., when no perturbations occurred)**.

Group	Total 3-day periods	Downward heuristic	Close competitor	Bullying	No. shifts after removals	No. shifts after reintroductions	No. shifts after perturbation	No. shifts stabilization period
2020 (non-experimental group)	3	–	–	3 (100%)	–	–	–	0
2021 non-experimental phase	10	10 (100%)	–	–	–	–	–	0
2021 experimental phase	12	6 (50%)	–	6 (50%)	1	3	4	3
2022-1 (experimental group 1)	26	9 (35%)	11 (42%)	6 (23%)	3	2	5	6
2022-2 (experimental group 2)	26	10 (38.5%)	6 (23%)	10 (38.5%)	3	4	7	7
Total	77	35 (45%)	17 (22%)	25 (33%)	7	9	16	16

**Fig 3:**
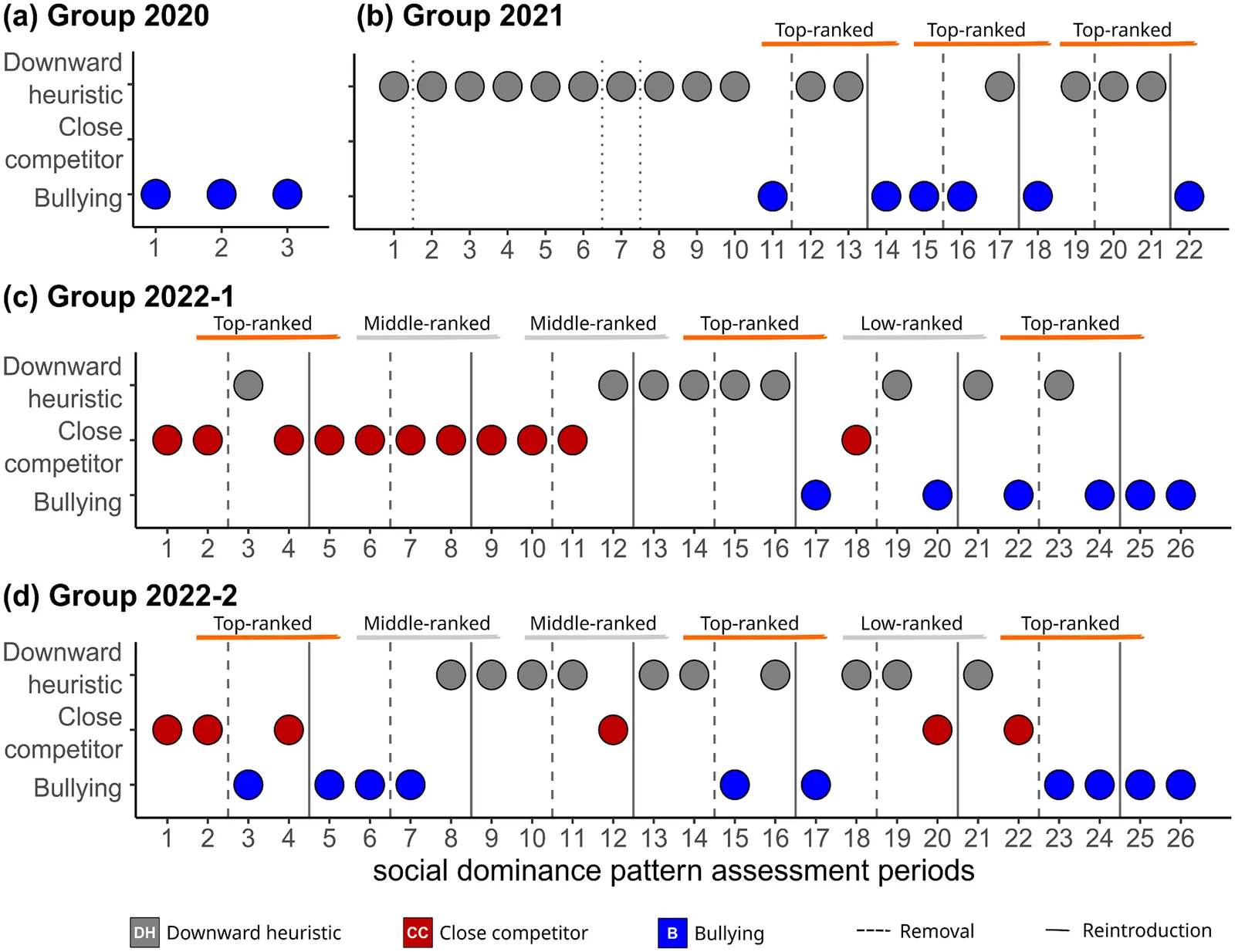
Dominance patterns primarily changed over time when group composition was experimentally perturbed. Dominance patterns are an emergent group-level pattern where group members make similar rank-informed aggression decisions. Each panel represents a different social group with the dominance patterns for each assessment period. The dotted lines in panel (b) represent nonexperimental perturbations (a thunderstorm, removal of 2 injured birds, and finally a dominant partner switch). Perturbations consisted of removal (dashed line) and reintroduction (solid line) of a top-ranked (orange highlight) or lower-ranked (either middle- or low-ranked, grey highlight) focal bird.

When we performed our perturbation experiments, we detected a total of six dominance pattern shifts in our 2021 experimental phase. The group followed a bullying pattern in 50% of the assessment periods and the downward heuristic pattern in the other half of the assessment periods (Table 3). Four of the six shifts occurred directly following a perturbation (Fig 3b). We found that dominance patterns in our two 2022 groups also changed over time. Both groups switched between all three possible dominance patterns but exhibited different propensities to follow each dominance pattern (Table 3, Fig 3c, 3d). We detected a total of eleven dominance pattern shifts in group 2022–1 and five of those occurred directly following a perturbation. In group 2022–2, we detected 14 shifts of which seven occurred directly following a perturbation.

### Dominance rank effects on aggression dynamics and pattern shifts

We first analyzed the change in aggression balance between the periods before removal and upon reintroduction across the experimental groups using a robust mixed model. Aggression balance change varied with focal rank, focal status, and their interaction. Birds (both focal birds and group members) in top-ranked perturbations (0.03 ± 0.03, [-0.98-0.78], n = 126) showed a greater change in aggression balance than birds in lower-ranked perturbations (-0.02 ± 0.03, [-0.99-0.48], n = 66; β = 0.68, 95% CI [0.47, 0.88]; Fig 2b). As well, focal birds showed a much greater change in aggression balance (mean ± SE = -0.45 ± 0.11, range [-0.98-0.09], n = 15 birds) than the remaining group members (0.05 ± 0.02, [-0.99–0.78], n = 177 birds); β = 0.97, 95% CI [0.84, 1.11]; Fig 2b). The interaction between focal rank and focal status was significant and negative (β = -0.76, 95% CI [-0.97, -0.54]; Fig 2b), indicating that the difference between focal birds and group members was smaller in lower-ranked than in top-ranked perturbations. We observed more aggressive interactions overall during top-ranked (461.6 ± 81.0, [96–1110], n = 18) compared to lower-ranked perturbations (247.1 ± 38.9, [90–478], n = 12; LR test: λ = 7.65, P = 0.02; Fig 2c). Results from a binomial mixed model on raw aggression counts were qualitatively consistent with those from the robust mixed model on change in aggression balance: focal birds showed a stronger reduction in aggression after reintroduction than nonfocal birds, and this difference was greater in top-ranked than in lower-ranked perturbations (Table D in S1 Appendix).

To test if the group responded differently to individual birds depending on their rank, we compared the total number of aggressive interactions received between the focal birds upon reintroduction (reintroduced birds) and similar ranked birds prior to the focal’s reintroduction. We found that the rank of the focal bird resulted in different aggression responses. Reintroduced birds received more aggression (mean ± SE = 533.2 ± 83.9, range [143–1110], n = 15) than similarly ranked birds (218.4 ± 38.7, [90–640], n = 15; LR test: λ = 15.65, P < 0.001; Fig 2c). We found no interaction effect for received aggression between focal rank and focal status (LR test: λ = 1.99, P = 0.16; Fig 2c) suggesting that reintroduced birds and group members received aggression in similar ways across top-ranked and lower-ranked perturbations. When we analyzed whether the number of aggressors differed between reintroduced birds and similarly ranked birds, we found that both were aggressed against by most group members. Reintroduced birds and similarly ranked birds received aggression from a similar number of distinct birds, with the proportion controlled for by group size averaging 0.82 ± 0.02 (SE) (range: [0.6-0.95], n = 30; Fig D in S1 Appendix).

In our two 2022 groups, we performed three top-ranked and three lower-ranked perturbations, allowing us to compare the number of shifts (n = 6 total possible shifts) and the use of dominance patterns across focal rank in these groups. Top-ranked perturbations resulted in 3 out of 6 shifts compared to shifts in 2 out of 6 lower-ranked perturbations in group 2022–1. In group 2022–2, we detected 5 out of 6 shifts after top-ranked perturbations and 2 out of 6 shifts after lower-ranked perturbations. Across both groups combined, we found that top-ranked perturbations resulted in more dominance pattern shifts (8/12, 67%) compared to lower-ranked perturbations (4/12, 33%; Fig 4) but this difference was not significant (one-sided Fisher’s exact test: odds ratio = 4.0, P = 0.11).

**Fig 4:**
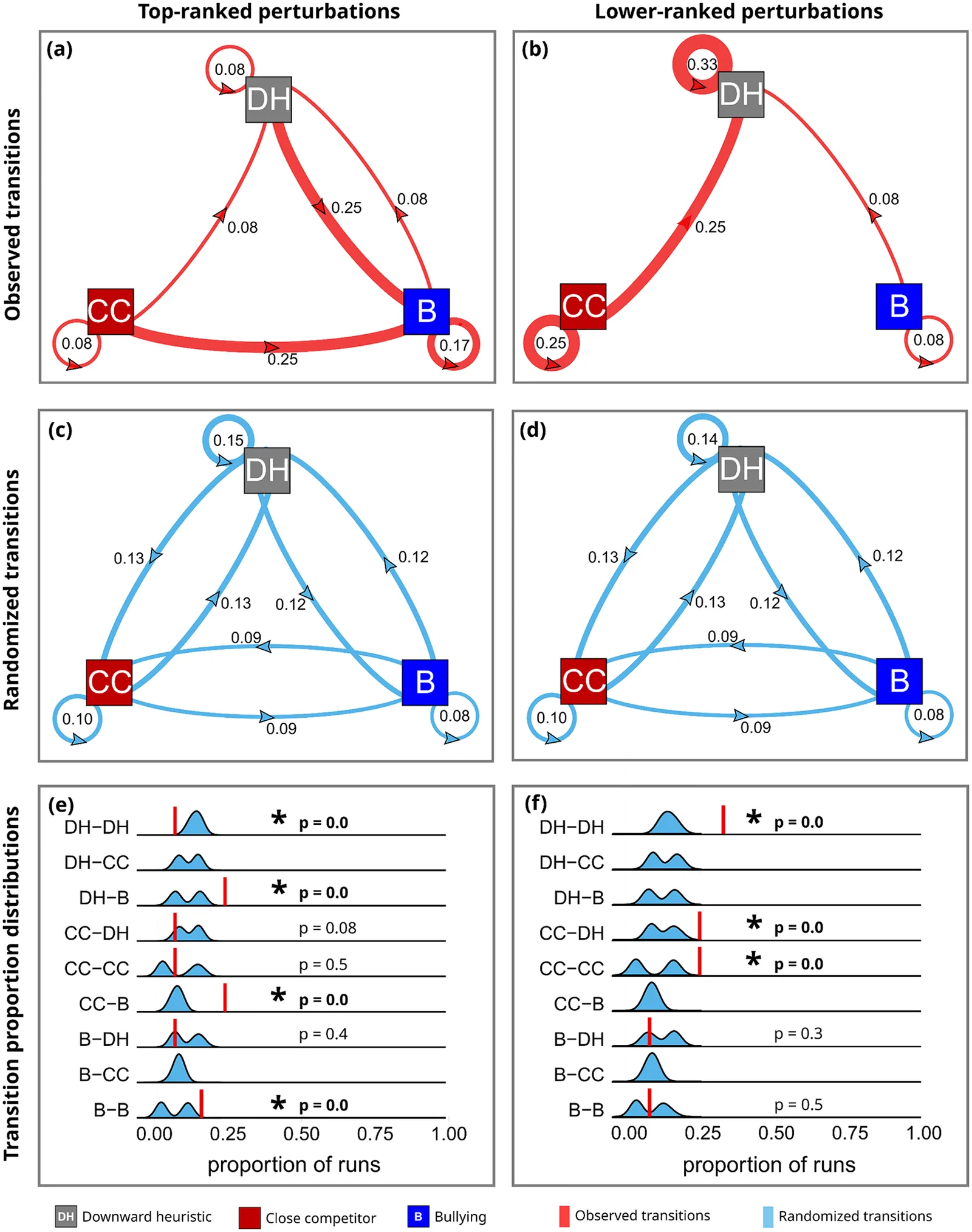
Perturbations of top-ranked monk parakeets resulted in different shifts than perturbations of lower-ranked birds. Using transition diagrams, we summarized dominance pattern transitions in the groups studied in 2022 following perturbations (removals and reintroductions combined) of (a) top-ranked and (b) lower-ranked focal birds. The proportions represent the number of observed transitions divided by the total number of perturbations of that rank type. Panels (c) and (d) show the corresponding average transition proportions from randomized dominance patterns across all six top-ranked and lower-ranked perturbations, respectively. The bottom panels show the distributions of the proportion pattern changes of (e) top-ranked and (f) lower-ranked perturbations. The y-axis shows the number of runs (out of a 1000) in which a given pattern transition occurred in at least one perturbation. We included in bold the proportion of reference values less than the observed value and in black the proportion greater than the observed value. If the observed patterns fall outside the distribution of changes produced by the reference model, this provides evidence that focal rank could be important in describing the observed shifts. In contrast, if the observed patterns fall within the reference model distribution, the shifts could arise from random processes rather than focal rank.

While we found no evidence that the number of shifts differed, we did find evidence that the groups transitioned between dominance patterns differently dependent on the rank of the focal bird. We found that the groups switched to or kept a bullying pattern in 8 out of 12 (67%) top-ranked perturbations (Fig 4a). When we compared these shifts to expectations if dominance pattern changes were randomly ordered (Fig 4c, 4e), we found evidence that the shifts from a downward heuristic to a bullying pattern, from a close competitor to bullying, and when patterns remained a bullying pattern happened more often than expected by chance and the difference between the observed and randomized transition proportions was 0.13, 0.16, and 0.09, respectively (Fig 4e). Contrastingly, the groups keeping a downward heuristic pattern happened less often than expected by chance with a difference of -0.07 (Fig 4e). In lower-ranked perturbations, group members primarily directed their aggression toward anyone ranked lower than themselves (shift to or kept a downward heuristic pattern in also 67% of perturbations; Fig 4b). We found evidence that the shift from a close competitor to a downward heuristic pattern and that the pattern remained a close competitors or a downward heuristics pattern happened more often than expected by chance with differences between observed and random transition proportions of 0.12, 0.15, and 0.19, respectively (Fig 4d, 4f). We included the results by rank and perturbation type in Fig F in S1 Appendix and summarized the dominance pattern transitions across all perturbations and social groups (Fig G in S1 Appendix). Focal rank did not affect the number of pattern shifts but did affect what dominance pattern was used by the group after top- or lower-ranked perturbations. The groups primarily switched to or kept a bullying pattern in top-ranked perturbations and the groups primarily switched to or kept a downward heuristics pattern in lower-ranked perturbations.

### Perturbation type effects on aggression dynamics and dominance patterns

As focal birds were absent in the periods after removal and before reintroduction, we analyzed the change in aggression balance before vs after removals and before vs after reintroductions only for the remaining group members in the two 2022 groups using a robust mixed model. Reintroduction increased aggression balance change relative to removals (removal 0.03 ± 0.02, n = 177; reintroduction 0.06 ± 0.01, n = 177; β = 0.06, 95% CI [0.01, 0.10]; Fig 2d), but this effect was significantly weaker in top-ranked perturbations (interaction β = −0.07, 95% CI [−0.12, −0.01]; Fig 2d). Top-ranked perturbations showed higher change than lower-ranked perturbations (top-ranked 0.05 ± 0.01, n = 234; lower-ranked 0.02 ± 0.01, n = 120; β = 0.094, 95% CI [0.05, 0.14]; Fig 2d). Individual-level variance was estimated as zero, indicating no detectable variation in baseline change across individuals after accounting for fixed effects. Group-level variance was small (SD = 0.03), suggesting limited differences between groups. Residual variance (SD = 0.13) accounted for most of the unexplained variability.

Because we found a strong difference in how the groups transitioned dominance patterns after top-ranked perturbations compared to lower-ranked perturbations (Figs 3, 4, F and G in S1 Appendix), we investigated the difference in response after removals and reintroductions for the top-ranked perturbations only. Across the top-ranked perturbations in all three experimental groups, we observed a similar number of shifts prior to and after removals and reintroductions (Fig G in S1 Appendix). In both cases, we observed shifts in a total of 6 out of 9 perturbations (67%; odds ratio = 1, P = 0.7). Although this result showed that the number of shifts in removals compared to reintroductions was not significantly different, we did find a difference in the ways dominance patterns transitioned from pre-perturbation to post-perturbation when we compared removals and reintroductions (Fig 5a, 5b). After removal, we found that in 5 out of 9 perturbations, the dominance pattern shifted to or remained a downward heuristic pattern (Fig 5a). After reintroduction, the dominance pattern shifted to or remained a bullying pattern in 8 out of 9 perturbations (Fig 5b). When we compared the shifts to expectations if dominance pattern changes were randomly ordered (Fig 5c, 5d), we found evidence that the shift from a close competitor to a bullying pattern happened more often than expected by chance after removal and the difference between the observed and randomized transition proportions was 0.14 (Fig 5e). After reintroductions, we found evidence that the shift from a downward heuristic to a bullying pattern and that the pattern remained a close competitors pattern happened more often than expected by chance (Fig 5f). The difference between the observed and randomized transition proportions for the shift from downward heuristic to bullying and from close competitors to close competitors after reintroduction, was 0.39 and 0.08, respectively. Perturbation type did not affect the number of pattern shifts but did affect what dominance pattern was used by the group after each perturbation of a top-ranked bird, where, particularly, the shift from downward heuristic to bullying upon reintroduction was significantly different from a random distribution.

**Fig 5:**
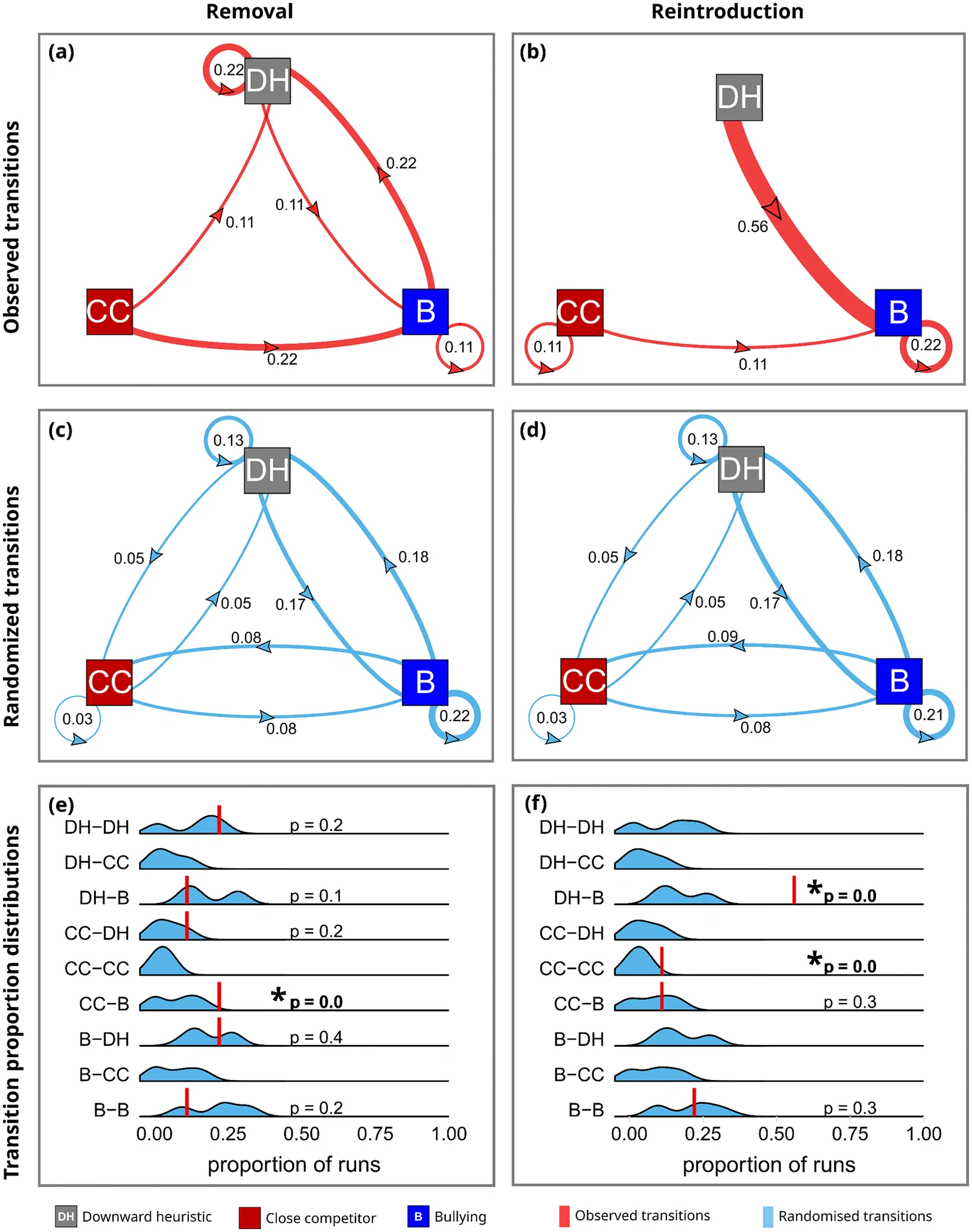
Reintroductions resulted in different shifts compared to removals across all nine top-ranked perturbations. We show transition proportions of the observed dominance patterns (a) after removal and (b) upon reintroduction, and the average transition proportions of the randomized dominance patterns (c) after removal and (d) upon reintroduction. The bottom panels show the distributions of the proportion pattern changes (e) after removal and (f) after reintroduction. The y-axis represents the number of runs (out of 1000) in which a given pattern transition occurred in at least one perturbation. We included the proportion of reference values that was less than the observed values in bold and the proportion of reference values that was more than the observed values in black. If the observed patterns fall outside the distribution of changes produced by the reference model, this provides evidence that perturbation type could be important in describing the observed shifts. In contrast, if the observed patterns fall within the reference model distribution, the shifts could arise from random processes and not the perturbation type.

## Discussion

We assessed social plasticity in captive parakeet groups by determining whether individual aggression decisions and dominance patterns changed over time within social groups, social perturbations could induce such changes, and the focal bird’s rank and the type of perturbation (removal vs. reintroduction) affected changes in aggression and dominance patterns. We found that individual aggression decisions could change over time and groups could shift their dominance patterns. We also found that these changes were associated with our experimental perturbations, and both the rank of the removed birds as well as whether the perturbation was a removal or a reintroduction affected aggression and how groups transitioned between dominance patterns, but not the total number of transitions. Our results highlight that groups subjected to experimental manipulations of social conditions led to predictable changes in aggression dynamics and dominance patterns. These findings also provide evidence that captive monk parakeet groups were capable of social plasticity.

### Dominance patterns shifted over time within groups

Our results across multiple replicate groups provide evidence that monk parakeet groups shifted between dominance patterns over time. We documented 29 shifts in dominance patterns, with different groups showing multiple shifts over time, confirming that dominance patterns can be plastic features of a social group. Treating social traits as inherent to a species is based on the idea that sociality depends mostly on the phylogenetic history of a species, rather than representing an adaptive response to changing socioecological conditions [24]. However, if a group can adaptively respond to changes in socioecological conditions, we would expect to observe variability not only across social groups of a given species, but within social groups over time. Groups that can respond to changes in conditions by altering their behavior and switching to a different dominance pattern may be more resilient to short term disruptions [25–27]. We found that many of the dominance pattern shifts were observed directly following a change in group composition. While most shifts followed perturbations, they did not exclusively occur after a perturbation, and not all perturbations resulted in shifts.

### Rank and perturbation type affected overall aggression and the types of dominance shifts

Our experiments showed that aggression dynamics from the group to the focal bird, as well as the group’s overall dominance pattern, both responded to our rank-based perturbations. Particularly, the response to top-ranked perturbations and reintroductions were different compared to lower-ranked perturbations and removals.

For aggression dynamics, the number of aggressive events received by top-ranked focal birds upon reintroduction was much higher compared to the amount of aggression received by lower-ranked birds upon reintroduction. We also found that these changes could not be attributed to simple rank changes of the focal birds, as aggression from the group towards rank-matched birds prior to reintroduction was different from aggression towards the focal once it had rejoined the group. These findings are consistent with previous results showing that rank determinants in monk parakeets are more based on the history of social interactions than on individual characteristics [13,28]. Additionally, the observed differences in the change in aggression received and given (aggression balance) suggests that aggression was more dependent on the identity of the focal bird that occupied a certain rank position, than the rank position itself.

For dominance patterns, we had predicted that top-ranked perturbations and removals would result in more pattern shifts than lower-ranked perturbations and reintroductions, respectively. However, in our parakeet groups, we found no significant difference in the number of dominance pattern shifts. This finding suggests that both focal rank and perturbation types could change the group’s information and either force, or allow the opportunity for, the remaining group members to update their rank.

While we found no evidence that the number of shifts differed, we did find evidence that the rank of the perturbed bird affected the pattern the group shifted to. Even though not all perturbation events triggered a dominance pattern shift, the perturbations of top-ranked birds (key individuals) made up most perturbations that triggered a shift. Other animal groups are also susceptible to perturbations of top-ranked individuals [29–34]. For example, in pig-tailed macaques (*Macaca nemestrina*), key individuals manage conflict within groups and their removal results in overall destabilization of the social structure [29,30]. Similarly, targeted removals of key individuals compared to random individuals in killer whale (*Orcinus orca*) networks resulted in fragmented groups, which disrupts social structure and potentially threatens population viability [31]. In our monk parakeet groups, the perturbation of key individuals affected individual aggression decisions, and influenced the direction of dominance pattern shifts.

Our experiments also provide potential insight into how perturbation type affected the pattern the group shifted to. Shifts in dominance patterns after key individual removal may suggest there was a power vacuum and remaining groups members changed their aggression to seize the opportunity to update their ranks [35]. Perhaps during the reintroduction, social history of past interactions was still important and the group members wanted to keep their updated rank and did not want to lose it again to the reintroduced bird [13].

The effect of removing individuals from social groups has been extensively studied in several species [30–32,36–41]. The introduction of new individuals through birth and immigration or the return of absent group members has received less attention as these processes in wild populations are harder to follow (but see [37,42]). Our study now highlights that the reintroduction of individuals can potentially have group-level consequences on hierarchical organization and dominance patterns. However, it is important to note that a direct comparison between removal and reintroduction dynamics here should be done carefully and with caveats due to a difference in our handling and trapping methods between removals (all birds captured) and reintroductions (just reintroduced bird handled).

### Relating our results to the wild

Our experimental approach identified the range of responses captive monk parakeet groups exhibited following social perturbations, where removals could reflect death or dispersal of a group member [5], and reintroduction the return of a temporary absent group member due to fission-fusion dynamics [43]. However, just because a species can respond in a certain way during controlled captive experiments does not guarantee that wild populations will respond similarly. Understanding how wild groups would respond would require similar experiments in wild populations. Future work could therefore include comparative studies in wild monk parakeets, as well as in species with similar social organization, specifically those with long-lasting preferred relationships in communal roosts and high fission-fusion dynamics during daytime foraging, e.g., sulphur-crested cockatoos (*Cacatua galerita* [44,45]), orange-fronted conures (*Aratinga canicularis* [46]), Bechtein’s bats (*Myotis bechsteinii* [47]), and ravens (*Corvus corax* [48,49])*.* Research on wild populations may also be able to provide insight into the long-term possible benefits of changes in dominance patterns. More broadly, studies in other species would also be essential in determining how widespread these shifts in dominance patterns might be and whether species may predictably shift in a certain way following perturbations. Overall, a group’s response to the perturbation of a group member may be species- and context-dependent [29–32,36–40], where the social system and environment (e.g., resource availability [33]) may influence whether a species is resilient to a perturbation.

Our experimental design (stable groups, single-individual removals for 8 days, and controlled reintroduction into the same group) does not necessarily resemble what individuals experience in the wild. For example, we chose a removal and reintroduction period of 8 days to obtain a balance in providing the birds ample opportunity to restabilize their social structure and obtaining sufficient perturbation replication. Wild monk parakeets exhibit fission–fusion dynamics across several dimensions, including variation in flock size, spatial cohesion, and duration of fission-fusion events [15]. Wild flocks can range from one to 95 birds, with a mean of approximately four birds, and flock size varies with activity [16]. Thus, individuals in the wild may experience more frequent and variable changes in the number and identity of their associates and duration of separation than occurred in our experiment. Nevertheless, the perturbation of a single bird in our study was sufficient to alter aggression dynamics and trigger shifts in dominance patterns. These results should not be interpreted as a direct estimate of the response of wild populations. Rather, they demonstrate under controlled conditions that changes in group membership can reorganize aggression at the group level and generate testable predictions for wild systems. The variability that species with fission-fusion dynamics can experience in the wild may make their social structure more flexible and accustomed to frequent group membership changes than our captive groups. Alternatively, repeated changes may reduce the effect of any single perturbation. Ongoing work in our study population is examining whether experimentally imposed fission–fusion dynamics that more closely mimic natural variation in group size, membership, and association duration also produce changes in aggression dynamics and dominance patterns.

### Potential limitations and future directions

It is important to note that responses to 1) removals and reintroductions, and 2) top-ranked versus lower-ranked perturbations should be compared with caveats. First, on removal days, all birds were trapped and handled, but on reintroduction days, only the reintroduced bird was handled to minimize stress [50–52] by reducing the number of trapping events for the group. Because of differences in methods, we could not fully separate treatment effects from possible effects of the trapping procedure itself, and it remains possible that handling stress contributed to some of the observed changes in aggression. However, since we analyzed aggression excluding the trapping day, the birds should have had sufficient time to recover from handling stress. Capture and handling typically elicit a strong but short-lived corticosterone response, with levels returning toward baseline within an hour and no consistent evidence of long-term behavioral disruption when animals are released promptly [51–53]. We also found that lower-ranked reintroductions were not commonly associated with a switch to a bullying pattern; if differences in trapping and handling alone was the primary cause of a change in dominance pattern on reintroduction, we would have expected to see similar shifts in both the top-ranked and lower-ranked reintroductions. We also have some evidence that disturbance alone was unlikely to directly cause dominance pattern shifts: we experienced two unplanned disturbances during the 2021 field season that disrupted behavior, group composition, and dominance hierarchy, but none of these perturbations resulted in shifts in dominance patterns. The patterns we observed can therefore be reasonably, but not certainly, attributed to differential responses to the perturbation type (removals versus reintroductions) rather than just differences in handling. Nevertheless, we acknowledge that our study design does not provide a clean control for the trapping procedure itself, and that this remains an important alternative explanation. To clearly differentiate between these effects would require two additional experiments in which (i) the entire group is captured and released on both removal and reintroduction days, and (ii) capture and release of the entire group is performed without experimental perturbations.

Second, differences between perturbations involving top- and lower-ranked individuals may reflect not only the characteristics of the removed bird, but also the altered composition of the remaining group. When a top-ranked bird is removed, the remaining group consists entirely of lower-ranked individuals, whereas removing a lower-ranked bird leaves the top-ranked individual and most of the original hierarchy intact. Consequently, differences in group responses between these treatments could reflect either the disproportionate influence of losing a (key) individual or the different social contexts experienced by the remaining members. Our design does not allow these two effects to be completely separated. Disentangling these effects would require study designs that manipulate group composition independently of the focal individual’s rank, for example by removing multiple individuals simultaneously or by swapping individuals between groups.

### Possible adaptive benefits of social plasticity in dominance pattern dynamics

This study is the first to document that a group can shift their dominance patterns over time, but the ways this might benefit individuals in a group is still an open question. One potential explanation for groups to shift their dominance pattern is when individuals obtain benefits by maintaining or gaining rank. Interestingly, we did not observe a consistent shift towards a close competitors pattern, even though using this pattern may help individuals preserve their own ranks. For example, it might be beneficial for individuals to switch to a close competitors pattern if the perturbation leads to conditions that are conducive to rank overthrow because this pattern could reduce the chances of rank challenges from close-ranked opponents. Female gorillas (*Gorilla* spp.) direct their aggression to others close in rank to reinforce their status, particularly with heightened competition when there are more females in the group [54]. In our monk parakeet groups, we observed that the two larger groups never used a close competitors pattern, while the smaller groups did (33% of 3-day periods). Thus, group size may not necessarily translate to competition in our study. Alternatively, individuals may benefit from shifting to a downward heuristic pattern in times of social upheaval if aggression across all lower-ranked individuals helps re-stabilize the structure of the hierarchy, or if individuals are susceptible to rank overthrows from any lower-ranked challenger.

The shifts towards bullying a previously top-ranked reintroduced individual, which occurred the most in this study, may be beneficial for group members as it may allow group members to preserve their new rank status. Upon reintroduction, the remaining birds in the group could work together to suppress the ability of the formerly top-ranked bird to re-take the top rank. Other factors might also explain the switch to bullying, such as copying other group member’s behavior [55]. Our results contradict simple copying of aggressive targets because we observed a difference between how top and lower-ranked birds were targeted following their reintroductions. A switch to bullying can also occur if individuals use aggression to signal dominance to potential opponents observing aggression [56] or to deter potential opponents from aggressing [57]. Previous work with monk parakeets has provided strong evidence that the parakeets remember the identities of opponents, outcomes of their own fights, and the opponents and outcomes of others [28] and that rank in these groups appears to be an outcome of social history in the groups [13]. Thus, there is a potential basis for thinking of aggression and bullying in these parakeets as a signaling system rather than being solely an outcome of competition for resources. Other work found that bullying may be involved in information transfer. For example, captive common waxbills (*Estrilda astrild*) showed a bullying pattern particularly when the audience consisted of waxbills that were not close associates of the aggressors [56]. Also, in paper wasps (*Polistes dominulus*) aggression functioned as a deterrent signal, where wasps used short-term social history and memory for aggressive decision-making [57]. Thus, bullying may be a method for individuals to signal their rank to uninformed individuals or potential opponents.

## Conclusions

Using social perturbation experiments, we provide evidence that monk parakeets showed social plasticity in structured aggression in response to changing social environments. Our results showed that individuals adjusted their aggression decisions, that a single group could change their dominance pattern use over time, and that the propensity to shift appeared to be mainly, but not exclusively, associated with changes in group membership. Many animal species face changes in the social and physical environment through both internal and external factors, such as naturally occurring demographic processes or through threats associated with urbanization and climate change [5,58,59]. Social plasticity may be one way for species to show resiliency to changing environments [25,26].

## Methods

### Ethics statement

The University of Cincinnati IACUC protocol (#AM02-19-11-19-01) and the National Wildlife Research Center Quality Assurance (#3203) approved all animal-related activities.

### Social groups

We observed four captive groups of monk parakeets at the United States Department of Agriculture, Wildlife Services, National Wildlife Research Center (USDA WS NWRC), in Gainesville, Florida. The four groups differed in group size, size of the flight pen, tenure in captivity, the time of year we observed the groups, and the experimental conditions. To answer whether dominance patterns shift when group composition was not perturbed, we performed behavioral observations of one captive social group from March until April 2020 and another captive group from March until April 2021 (Table A in S1 Appendix). Both groups were introduced into a large 45 x 45 m flight pen.

In 2020, we observed a group of 20 monk parakeets that were long-term captives. USDA personnel captured these birds from four different feral populations in Southern Florida in 2003, 2007, and 2012. Three of the 20 parakeets were hatched in captivity in 2006 and 2007 at the USDA WS NWRC facility. We observed for a total of 191.3 person hours across 12 days, with an average of 20.5 ± 3.6 (SD) hours of observation per three-day assessment periods (n = 3 periods). The observation period was much shorter than planned due to disruptions from the COVID-19 pandemic, which required us to drastically truncate our field season.

In 2021, we observed a group of 22 monk parakeets captured from four feral populations in Southern Florida in February 2021 just prior the experiment. We observed for a total of 832.8 person hours across 31 days, with an average of 22.1 ± 2.4 (SD) hours of observation per three-day assessment period (n = 10 periods). We allowed the group to interact to stabilize their dominance hierarchy prior to our perturbation experiment. This initial stabilization period lasted 31 days as we incurred unplanned perturbations. There was a thunderstorm which resulted in the removal of two injured birds (both lower-ranked) to receive professional care after our first capture event. The other unplanned perturbation was a partner switch of the most dominant bird which caused the dominance hierarchy to destabilize. We considered that these events were not part of our planned perturbation experiment.

To answer whether the removal and reintroduction of a top-ranked bird could change the dominance patterns, we observed the same social group of 20 birds that was already present in the large flight pen for 31 days in 2021 (see paragraph above, Table B in S1 Appendix). We performed the perturbation experiment from May until July 2021 and we started the perturbation experiment after the birds had interacted for 31 days in the flight pen. In total, we observed over 940.3 person hours in 37 days, with an average of 21.3 ± 2.3 (SD) hours of observation per three-day assessment period (n = 12 periods, from period 11 onward).

From January through May 2022, we performed the perturbation experiments in two groups of eleven monk parakeets that were caught from feral populations in 2021 (Table C in S1 Appendix) and consisted of birds previously (n = 14 birds) and not previously (n = 8 birds) used in our 2021 experiment. Four observers monitored the groups from different blinds in two 10 x 4.5 x 3m flight pens. Two observers focused on one social group each day and the observers were randomly assigned to one of the four blinds. In total, we observed over 793.9 person hours in 77 days, with an average of 17.6 ± 3.2 (SD) hours of observation per three-day assessment period (n = 26 periods). In group 2, we observed for 771 person hours in 78 days, with an average of 17.9 ± 3.7 (SD) hours of observation per three-day period (n = 26 periods).

### Social interaction data collection

Multiple observers (3–4 observers) performed daily observations approximately between 08:00 and 19:00 from different blinds. The observers recorded aggressive dyadic interactions using all-occurrence sampling [60] using the Animal Observer application (Diane Fossey Gorilla Fund v1.0 [61],). Observers identified the birds using a unique body color combination made with nontoxic permanent markers (Sharpie, Inc.) [62]. We recorded aggressive interactions with a clear winner (decided aggression events [63]). The recorded aggressive interactions included crowds (the aggressor approaches a target, but the target moves away before the aggressor is in striking range) and displacements (the aggressor aggressively approached another bird within striking range and supplanted it from its location). We used a previously described framework to ascertain that crowds and displacements were functionally similar and thus part of the same behavioral context [18]. We combined crowds and displacements for further analyses, removed duplicate observations, and retained the interactions where both the actor and the receiver were identified (see for details [13]).

### Rank-based removals

To start our perturbation experiments, we calculated the dominance rank of all group members using aggressive interaction events where there were clear winners and losers binned across three days of observations prior to removals and a modified version of PageRank, called power score, using the ‘Domstruc’ package [7,13,23]. Only one of the observers (AM) performed the dominance rank analyses, the other observers were blind to the standing of the remaining group members in the dominance hierarchy. In our 2021 experiment, we performed removal/reintroduction perturbations of three top-ranked birds (termed top-ranked perturbations). In 2022, we performed perturbations of three top-ranked, two middle-ranked and one low-ranked bird per group. We combined the latter two into a lower-ranked perturbation category as comparison to the top-ranked perturbations. We randomized the order of the perturbations prior to the start of the field season.

To remove the focal bird, we caught all birds in the morning using mist nets and removed the focal. We placed the focal back in its standard housing cage (2 x 2m wire cage). The focal bird was by itself in the housing cage but this cage was positioned in larger housing with other parakeets away from the experimental group in the flight pen. We then released the remaining birds back into the flight pen. We did not use the observations of the trapping day for our analyses to allow the birds to recover from the stress of capture and so that our results were less likely to be affected by any immediate effects of handling. We allowed the remaining group members to interact undisturbed for eight days which is sufficient time for the social structure to restabilize [62]. We reintroduced the removed bird at the same time (8:30) and location each time after the 8-day removal period. We recorded observations immediately after the reintroduction of the focal to investigate the group’s response to the reintroduced bird. We then allowed the birds to interact for another seven days. In the middle of each removal and reintroduction period, the observers had a 2-day break. This schedule resulted in three days of observations immediately before and after removals and reintroductions, with one extra day in the removal phase which was the day we trapped all birds and removed the focal bird. We binned all aggressive interactions within these 3-day periods to perform our analyses. We used the aggressive interactions binned across the three days of observations prior to a removal to identify the focal for the perturbation (Tables B and C in S1 Appendix).

### Dominance pattern assessment

We used the aggressive interaction events binned across three days of observations to assess the dominance patterns. We used the function *domstruc* from the ‘Domstruc’ package [23] to calculate the global dominance patterns [7]. This function uses two parameters. First, it calculates *focus*, which measures the distribution of the relative rank difference between the aggressor and the receiver. A value closer to 1 means that the receivers of aggression are all the same ranks down the hierarchy from the aggressors, while the focus will decrease when the aggression is more equally distributed across all ranks in the hierarchy. Second, the function calculates *position*, which reflects where aggression is most focused relative to the aggressor’s rank. Recent work has categorized rank-based group-level aggression into three categories [7]: 1) the downward heuristic, where individuals indiscriminately aggress against others ranked below themselves, 2) close competitors, where individuals preferentially aggress against those ranked slightly below themselves, and 3) bullying, where individuals preferentially aggress against those ranked far below themselves [7]. If the group follows a bullying pattern, the value of position would be closer to 1, whereas if the group follows a close competitor pattern, the value would be closer to 0.

To assess whether and which dominance pattern a group followed, the function compares the observed focus and position values to the values simulated by reference models. This reference model uses the observed rank order of the individuals in the group along with each bird’s total number of successful aggressive events, but re-assigns the targets of aggression by randomly choosing among the set of individuals ranked below the aggressor. To account for systems where aggression could move up the hierarchy instead of down the hierarchy as is defined for the downward heuristic pattern, a tuning parameter is implemented in the model. This tuning parameter is the fraction of aggressive interactions that are randomly allocated, because individuals could make mistakes or are opportunistic in their aggression decisions (see for example the rule breakers in Fig 2a). When the tuning parameter is 1, individuals aggress completely at random, whereas a value of 0 means that individuals aggress only individuals ranked lower than themselves. This model then shows how aggression within the sampling bin could be structured if the birds were forced to use a downward heuristic (with some noise) alone to structure their aggression. For each value of the tuning parameter, focus and position are estimated and the extremes of 95% confidence-interval error bars are used to draw a polygon around the region consistent with the downward heuristic pattern. We then compared the observed parakeet data to this distribution of values. If the observed focus and position parameters fall inside the polygon of the 95% CIs as the tuning parameter is varied from 0 to 1, we classified the group as having a downward heuristic pattern, because this would provide no evidence of additional structuring of aggression beyond what would be expected if the birds were just randomly targeting individuals ranked below themselves. If the focus and position values fell outside of the 95% CI of expected focus and position values, we categorized aggression as having more structure than expected based on simple use of a downward heuristic. If the observed focus and position fell above the polygon (higher relative rank difference), we categorized the aggression as bullying pattern, and if the quantities were lower, as a close competitors pattern. When focus values fall below those expected under a fully random system, patterns were considered undefined. This polygon was generated separately for each bin and for each group, meaning that similar observed focus and position values can be classified differently depending on group-specific structure.

### Statistical analyses

We performed all analyses in R version 4.1.2 [64] and created the figures using ggplot2 [65], diagram [66], and ggridges [67].

#### Aggression dynamics*.*

We first tested whether the number of aggressive interactions per 3-day assessment period stayed stable over time. We used a generalized linear mixed model (GLMM) for negative binomial distribution. We used the 3-day assessment period as fixed factor, group size as covariate and included an offset of hours observed and group ID as random factor. We used the package ‘glmmTMB’ [68] to implement the model, checked for overdispersion with the ‘performance’ package [69] and model diagnostics with the ‘Dharma’ package [70], and tested for significance using the likelihood ratio test [52].

For the experimental groups only, we analyzed how aggression given and received changed for individual birds. First, we constructed aggression networks using the ‘igraph’ package [58] as directed and weighted association matrices where the strength of the association was the total amount of aggressive events per dyad. As edges, we included the rule followers (amount of aggression that follows the dominance hierarchy where higher-ranking birds aggress against lower-ranking birds) and rule breakers (lower-ranked birds aggress against higher-ranked birds).

Next, we analyzed aggression balance (i.e., aggression given/[aggression given + received]), where a value of 1 means that the bird only aggressed, while a value of 0 means the bird only received aggression. We tested for the change in aggression balance for all birds between the pattern assessment period just before removal and upon reintroduction. A positive aggression balance indicates an increase in aggression, while a negative value indicates a decrease in aggression from before to after a perturbation. We analyzed the change in aggression balance with a robust linear mixed-effects model using the ‘robustlmm’ package [71], with focal rank (top-ranked or lower-ranked perturbation), focal status (focal bird or remaining group member), and their interaction as fixed effects and ID as a random intercept. The interaction establishes the aggression balance for focal birds in top-ranked perturbation or lower-ranked perturbations and group members in top-ranked and lower-ranked perturbations. This was in addition to testing for the difference in aggression balance for all perturbations (both top- and lower-ranked) between focal birds and remaining group members and the difference among all birds between top- and lower-ranked perturbations. We first also included group as random effect but that resulted in singular fit. Robust estimation was used to reduce the influence of outlying observations including those near the boundaries (the change in aggression balance is bounded between -1 and 1), thereby reducing their influence on parameter estimates, while preserving the repeated-measures structure of the data. In our data, most changes in aggression balance were intermediate rather than clustered at −1 or 1 (3% of absolute observations (6 out of 192) were above 0.9), suggesting that ceiling–floor effects were limited. Nonetheless, we verified that the results were qualitatively similar when modeling aggression counts (aggression given and aggression received) with a binomial mixed model. We reported the results of the robust mixed model and we interpreted the significance of the fixed effects using confidence intervals.

We then compared the total amount of aggression received for the focal birds upon reintroduction and birds that were similar ranked to the focal birds but in the period just prior to the focal’s reintroduction. We used GLMM for negative binomial distribution to compare number of aggressive events received for focal birds or similar ranked birds including an interaction with the rank of the focal bird. We included group ID as random factor. We used the ‘lme4’ package [72] to implement the model, checked model diagnostics with the ‘Dharma’ package [70], and tested for significance using the likelihood ratio test [73].

We also analyzed the number of distinct aggressors for focal birds and similar ranked birds using GLMM for beta distribution. We controlled for group size by dividing the number of aggressors by group size as the group size for the focal bird included one extra bird. We included an interaction effect between subject (focal bird or similar ranked bird) and focal rank (top-ranked vs lower-ranked perturbation), and group ID as random effect. We used the package ‘glmmTMB’ [68] to implement the model, checked model diagnostics with the ‘Dharma’ package [70], and tested for significance using the likelihood ratio test [73].

We finally analyzed the aggression balance change of the remaining group members (excluding the focal birds) between the periods before and after removal and before and after reintroduction to test for the effect of perturbation type on aggression dynamics. We used a robust linear mixed-effects model using the ‘robustlmm’ package [71], with perturbation type (removal or reintroduction), focal rank (top-ranked or lower-ranked perturbation), and their interaction as fixed effects, and group and ID as random intercepts.

#### Dominance pattern dynamics*.*

We summarized the dominance pattern changes between assessment periods using transition diagrams [66]. We included the results by removal and reintroduction and rank for the 2022 groups in Fig F in S1 Appendix. We then summarized the dominance pattern transitions across all perturbations and social groups to show the general patterns of the social manipulation experiment (for results see Fig G in S1 Appendix). To study how the groups respond to focal rank and perturbation type, we tested whether 1) the rank of the perturbed individual and 2) the perturbation type affected the propensity and types of group-level aggression changes. We kept these analyses separate as we used the two 2022 groups to study the response to focal rank, resulting in 6 top-ranked and 6 lower-ranked perturbations, and we used all top-ranked perturbations across all three experimental groups (2021, 2022–1, and 2022–2) to study the response to perturbation type, resulting in 9 removals and 9 reintroductions.

To study the propensity of dominance pattern shifts, we compared the proportion of dominance pattern shifts following 1) top-ranked versus lower-ranked perturbations and 2) removals and reintroductions using one-sided Fisher’s exact tests as we predicted that top-ranked perturbations and removals would result in more shifts compared to lower-ranked perturbations and reintroductions, respectively.

To study how the use of dominance patterns differed after 1) top-ranked and lower-ranked perturbations, and 2) removals and reintroductions, we analysed whether the observed pattern transitions prior and after the perturbations were different from random (Fig A in S1 Appendix). First, we randomized the order of the observed dominance patterns 1000 times (Fig A in S1 Appendix *step 1*). We then quantified the number of times the pattern remained the same or transitioned to another pattern across all 1000 reference models comparing the 3-day periods before and after 1) top-ranked and lower-ranked perturbations, and 2) removal and reintroduction perturbations, separately (Fig A in S1 Appendix *step 2a*). We calculated the proportion of randomized pattern transitions and averaged these across the perturbations, which we then visualized. Next, per reference model run, we summarized the number of perturbations that showed a particular dominance pattern change. We included all nine possible pattern changes per run and calculated the proportion of pattern changes per run across all perturbations. We visualized this reference distribution and included the observed pattern changes (Fig A in S1 Appendix *step 2b*). If the observed value falls outside the reference model distribution, this tells us that the observed change is unusual and is due to the perturbation. We determined whether observed values significantly differ from random values in the reference models using the proportion of random values that are less than the observed values. We used 2-tailed tests: observed values needed to be < 0.025 or >0.975 of values produced by the reference model to be considered significantly different.

## Supporting information

S1 Appendix List of Tables: Table A. The schedule of the experimental setup to study dominance patterns outside of experimental manipulations in monk parakeets. Table B. The 2021 experimental social perturbation schedule to study the effects of the perturbation of a key individual in monk parakeets. Table C. The 2022 experimental social perturbation schedule to study the response to the perturbations of differently ranked birds in monk parakeets. Table D. Sensitivity analysis of aggression balance. **List of Figures:** Figure A. A conceptual figure of the analysis to test how unusual the observed pattern changes are after perturbations. Figure B. The monk parakeet aggression networks are consistent throughout the social experiment in 2021. Figure C. Aggression balance over time. Figure D. Number of aggressors of focal birds and similar ranked birds. Figure E. Focus and position to categorize dominance patterns. Figure F. Dominance patterns and targeted aggression both changed differently depending on the rank of the perturbed individual and perturbation type. Figure G. Summary of the number of pattern transitions across all trials and experimental social groups.
(DOCX)

## References

[1] Ebensperger LA, Chesh AS, Castro RA, Tolhuysen LO, Quirici V, Burger JR, et al. Instability Rules Social Groups in the Communal Breeder Rodent *Octodon degus*. Ethology. 2009;115(6):540–54. doi: 10.1111/j.1439-0310.2009.01635.x

[2] Aguilar-melo AR, Calmé S, Smith-Aguilar SE, Ramos-Fernández G. Fission-fusion dynamics as a temporally and spatially flexible behavioral strategy in spider monkeys. Behav Ecol Sociobiol. 2018;72(150). doi: 10.1007/s00265-018-2562-y

[3] de Silva S, Ranjeewa ADG, Kryazhimskiy S. The dynamics of social networks among female Asian elephants. BMC Ecol. 2011;11:17. doi: 10.1186/1472-6785-11-17 21794147 PMC3199741

[4] Pinter-Wollman N, Hobson EA, Smith JE, Edelman AJ, Shizuka D, De Silva S. The dynamics of animal social networks: analytical, conceptual, and theoretical advances. Behav Ecol. 2013;25(2):242–55. doi: 10.1093/beheco/art047

[5] Shizuka D, Johnson AE. How demographic processes shape animal social networks. Behav Ecol. 2020;31(1):1–11. doi: 10.1093/beheco/arz083

[6] Taborsky B, Oliveira RF. Social competence: an evolutionary approach. Trends Ecol Evol. 2012;27(12):679–88. doi: 10.1016/j.tree.2012.09.003 23040461

[7] Hobson EA, Mønster D, DeDeo S. Aggression heuristics underlie animal dominance hierarchies and provide evidence of group-level social information. Proc Natl Acad Sci U S A. 2021;118(10):e2022912118. doi: 10.1073/pnas.2022912118 PMC7958391 33658380

[8] Shizuka D, McDonald DB. The network motif architecture of dominance hierarchies. J R Soc Interface. 2015;12(105):20150080. doi: 10.1098/rsif.2015.0080 25762649 PMC4387537

[9] Aplin LM, Farine DR, Mann RP, Sheldon BC. Individual-level personality influences social foraging and collective behaviour in wild birds. Proc Biol Sci. 2014;281(1789):20141016. doi: 10.1098/rspb.2014.1016 24990682 PMC4100518

[10] Sumpter DJT. The principles of collective animal behaviour. Philos Trans R Soc Lond B Biol Sci. 2006;361(1465):5–22. doi: 10.1098/rstb.2005.1733 16553306 PMC1626537

[11] van der Marel A, Waterman JM, López-Darias M. Barbary ground squirrels do not have a sentinel system but instead synchronize vigilance. Behav Ecol Sociobiol. 2021;75(11):153. doi: 10.1007/s00265-021-03094-1

[12] Strauss ED, Shizuka D. The dynamics of dominance: open questions, challenges and solutions. Phil Trans R Soc B. 2022;377(1845):20200445. doi: 10.1098/rstb.2020.0445 PMC8743878 35000440

[13] van der Marel A, Francis X, O’Connell CL, Estien CO, Carminito C, Moore VD. Perturbations highlight importance of social history in parakeet rank dynamics. Behav Ecol. 2023;34(3):457–67. doi: 10.1093/beheco/arad015

[14] O’Connell CL, Marel A van der, Hobson EA. The effect of familiarity on the temporal dynamics of spatial and affiliative associations in monk parakeets. Anim Behav Cogn. 2025;12(4).

[15] O’Connell CL, Carter GG, van der Marel A, Hobson EA. Monk parakeets “test the waters” when forming new relationships. Biol Lett. 2025;21(11):20250399. doi: 10.1098/rsbl.2025.0399 41218758 PMC12606181

[16] Hobson EA, Avery ML, Wright TF. The socioecology of Monk Parakeets: Insights into parrot social complexity. The Auk. 2014;131(4):756–75. doi: 10.1642/auk-14-14.1

[17] Hobson EA, John DJ, McIntosh TL, Avery ML, Wright TF. The effect of social context and social scale on the perception of relationships in monk parakeets. Curr Zool. 2015;61(1):55–69. doi: 10.1093/czoolo/61.1.55

[18] van der Marel A, Prasher S, Carminito C, O’Connell CL, Phillips A, Kluever BM, et al. A framework to evaluate whether to pool or separate behaviors in a multilayer network. Curr Zool. 2021;67(1):101–11. doi: 10.1093/cz/zoaa077 33654494 PMC7901760

[19] Modlmeier AP, Keiser CN, Watters JV, Sih A, Pruitt JN. The keystone individual concept: An ecological and evolutionary overview. Anim Behav. 2014;89:53–62. doi: 10.1016/j.anbehav.2013.12.020

[20] Drews C. The Concept and Definition of Dominance in Animal Behaviour. Behav. 1993;125(3–4):283–313. doi: 10.1163/156853993x00290

[21] Schjelderup-Ebbe T. Beiträge zur sozialpsychologie des haushuhns. Z Psychol. 1922;88:225–52.

[22] Tibbetts EA, Pardo-Sanchez J, Weise C. The establishment and maintenance of dominance hierarchies. Philos Trans R Soc Lond B Biol Sci. 2022;377(1845):20200450. doi: 10.1098/rstb.2020.0450 35000449 PMC8743888

[23] Mønster D, Hobson E, DeDeo S. R package version 0.1.0 [Internet]. domstruc: Compute Metrics of Dominance Hierarchies. 2021. Available from: https://github.com/danm0nster/domstruc

[24] Balasubramaniam KN, Dittmar K, Berman CM, Butovskaya M, Cooper MA, Majolo B. Hierarchical steepness and phylogenetic models: phylogenetic signals in *Macaca*. Anim Behav. 2012;83(5):1207–18. doi: 10.1016/j.anbehav.2012.02.012

[25] Testard C, Larson SM, Watowich MM, Kaplinsky CH, Bernau A, Faulder M, et al. Rhesus macaques build new social connections after a natural disaster. Curr Biol. 2021;31(11):2299-2309.e7. doi: 10.1016/j.cub.2021.03.029 33836140 PMC8187277

[26] Testard C, Shergold C, Acevedo-Ithier A, Hart J, Bernau A, Negron-Del Valle JE. Ecological disturbance alters the adaptive benefits of social ties. Science. 2024;384(6702):1330–5. doi: 10.1126/science.adk0606 PMC11995978 38900867

[27] Evans JC, Liechti JI, Boatman B, König B. A natural catastrophic turnover event: individual sociality matters despite community resilience in wild house mice. Proc Biol Sci. 2020;287(1926):20192880. doi: 10.1098/rspb.2019.2880 32370672 PMC7282912

[28] Hobson EA, DeDeo S. Social Feedback and the Emergence of Rank in Animal Society. PLoS Comput Biol. 2015;11(9):e1004411. doi: 10.1371/journal.pcbi.1004411 26355292 PMC4565698

[29] Flack JC, Girvan M, de Waal FBM, Krakauer DC. Policing stabilizes construction of social niches in primates. Nature. 2006;439(7075):426–9. doi: 10.1038/nature04326 16437106

[30] Flack JC, Krakauer DC, de Waal FBM. Robustness mechanisms in primate societies: a perturbation study. Proc R Soc B. 2005;272(1568):1091–9. doi: 10.1098/rspb.2004.3019 16024369 PMC1559816

[31] Williams R, Lusseau D. A killer whale social network is vulnerable to targeted removals. Biol Lett. 2006;2(4):497–500. doi: 10.1098/rsbl.2006.0510 17148272 PMC1834010

[32] Manno TG. Social networking in the Columbian ground squirrel, Spermophilus columbianus. Anim Behav. 2008;75:1221–8. doi: 10.1016/j.anbehav.2007.09.025

[33] Chaverri G. Comparative social network analysis in a leaf-roosting bat. Behav Ecol Sociobiol. 2010;64(10). doi: 10.1007/s00265-010-0975-3

[34] Piefke TJ, Bonnell TR, DeOliveira GM, Border SE, Dijkstra PD. Social network stability is impacted by removing a dominant male in replicate dominance hierarchies of a cichlid fish. Anim Behav. 2021;175:7–20. doi: 10.1016/j.anbehav.2021.02.012

[35] Bryden J, Silverman E, Powers ST. Status in flux: Unequal alliances can create power vacuums. arXiv [Internet]. 2019;1909.01826. Available from: http://arxiv.org/abs/1909.01826

[36] Naug D. Structure and resilience of the social network in an insect colony as a function of colony size. Behav Ecol Sociobiol. 2009;63(7):1023–8. doi: 10.1007/s00265-009-0721-x

[37] Firth JA, Voelkl B, Crates RA, Aplin LM, Biro D, Croft DP, et al. Wild birds respond to flockmate loss by increasing their social network associations to others. Proc R Soc B. 2017;284(1854):20170299. doi: 10.1098/rspb.2017.0299 28515203 PMC5443949

[38] Barrett L, Henzi SP, Lusseau D. Taking sociality seriously: the structure of multi-dimensional social networks as a source of information for individuals. Philos Trans R Soc Lond B Biol Sci. 2012;367(1599):2108–18. doi: 10.1098/rstb.2012.0113 22734054 PMC3385678

[39] Wiśniewska M, Puga-Gonzalez I, Lee P, Moss C, Russell G, Garnier S, et al. Simulated poaching affects global connectivity and efficiency in social networks of African savanna elephants-An exemplar of how human disturbance impacts group-living species. PLoS Comput Biol. 2022;18(1):e1009792. doi: 10.1371/journal.pcbi.1009792 35041648 PMC8797174

[40] Grossel LA, Javorouski ML, Moreira N, de Araujo Monteiro-Filho EL. Dominance hierarchy and social network in a captive group of white-lipped peccary males: what happens after the alpha male leaves? Acta Ethol. 2021;25(2):65–77. doi: 10.1007/s10211-021-00386-7

[41] Kubitza RJ, Suhonen J, Vuorisalo T. Effects of experimental perturbation of group structure on hierarchy formation and behaviour in House Sparrows. Ornis Fenn. 2015;92(4). doi: 10.51812/of.133877

[42] Boucherie PH, Gallego-Abenza M, Massen JJM, Bugnyar T. Dominance in a socially dynamic setting: hierarchical structure and conflict dynamics in ravens’ foraging groups. Philos Trans R Soc Lond B Biol Sci. 2022;377(1845):20200446. doi: 10.1098/rstb.2020.0446 35000442 PMC8743890

[43] Aureli F, Schaffner CM, Boesch C, Bearder SK, Call J, Chapman CA. Fission‐fusion dynamics: New research frameworks. Curr Anthropol. 2008;49(4):627–54. doi: 10.1086/586708 1558

[44] Penndorf J, Ewart KM, Klump BC, Martin JM, Aplin LM. Social network analysis reveals context-dependent kin relationships in wild sulphur-crested cockatoos *Cacatua galerita*. J Anim Ecol. 2023;92(1):171–82. doi: 10.1111/1365-2656.13839 36349451

[45] Aplin LM, Major RE, Davis A, Martin JM. A citizen science approach reveals long-term social network structure in an urban parrot, Cacatua galerita. J Anim Ecol. 2021;90(1):222–32. doi: 10.1111/1365-2656.13295 32629533

[46] Balsby TJS, Momberg JV, Dabelsteen T. Vocal imitation in parrots allows addressing of specific individuals in a dynamic communication network. PLoS One. 2012;7(11):e49747. doi: 10.1371/journal.pone.0049747 23185424 PMC3504101

[47] Kerth G, Perony N, Schweitzer F. Bats are able to maintain long-term social relationships despite the high fission-fusion dynamics of their groups. Proc Biol Sci. 2011;278(1719):2761–7. doi: 10.1098/rspb.2010.2718 21307051 PMC3145188

[48] Wright J, Stone RE, Brown N. Communal roosts as structured information centres in the raven, Corvus corax. J Anim Ecol. 2003;72(6):1003–14. doi: 10.1046/j.1365-2656.2003.00771.x

[49] Loretto MC, Schuster R, Itty C, Marchand P, Genero F, Bugnyar T. Fission-fusion dynamics over large distances in raven non-breeders. Sci Rep. 2017;7(1):380. doi: 10.1038/s41598-017-00404-4 PMC5428508 28336913

[50] Brandl HB, Farine DR. Stress in the social environment: behavioural and social consequences of stress transmission in bird flocks. Proc Biol Sci. 2024;291(2034):20241961. doi: 10.1098/rspb.2024.1961 39533955 PMC11558247

[51] van Oers K, Carere C. Long-term effects of repeated handling and bleeding in wild caught Great Tits Parus major. J Ornithol. 2007;148(2):185–90. doi: 10.1007/s10336-007-0200-y

[52] Le Maho Y, Karmann H, Briot D, Handrich Y, Robin JP, Mioskowski E, et al. Stress in birds due to routine handling and a technique to avoid it. Am J Physiol. 1992;263(4 Pt 2):R775–81. doi: 10.1152/ajpregu.1992.263.4.R775 1415787

[53] Watson H, Bolton M, Heidinger BJ, Boner W, Monaghan P. Assessing the effects of repeated handling on the physiology and condition of semi-precocial nestlings. Ibis. 2016;158(4):834–43. doi: 10.1111/ibi.12402 PMC5032895 27708454

[54] Smit N, Robbins MM. Risk-taking incentives predict aggression heuristics in female gorillas. eLife. 2025;14:RP107093. doi: 10.7554/eLife.107093 PMC12373394 40844092

[55] Deneubourg JL, Goss S. Collective patterns and decision-making. Ethol Ecol Evol. 1989;1(4):295–311. doi: 10.1080/08927014.1989.9525500

[56] Beltrão P, Gomes ACR, Cardoso GC. Bullying as an advertisement of social dominance in common waxbills. Proc R Soc B. 2023;290(2000):20230206. doi: 10.1098/rspb.2023.0206 PMC10265017 37312555

[57] Thompson FJ, Donaldson L, Johnstone RA, Field J, Cant MA. Dominant aggression as a deterrent signal in paper wasps. Behav Ecol. 2014;25(4):706–15. doi: 10.1093/beheco/aru063

[58] Blumstein DT, Hayes LD, Pinter-Wollman N. Social consequences of rapid environmental change. Trends Ecol Evol. 2023;38(4):337–45. doi: 10.1016/j.tree.2022.11.005 36473809

[59] Fisher DN, Kilgour RJ, Siracusa ER, Foote JR, Hobson EA, Montiglio P-O, et al. Anticipated effects of abiotic environmental change on intraspecific social interactions. Biol Rev Camb Philos Soc. 2021;96(6):2661–93. doi: 10.1111/brv.12772 34212487

[60] Altmann J. Observational study of behavior: sampling methods. Behaviour. 1974;49(3):227–67. doi: 10.1163/156853974x00534 4597405

[61] van der Marel A, O’Connell CL, Prasher S, Carminito C, Francis X, Hobson EA. A comparison of low‐cost behavioral observation software applications for handheld computers and recommendations for use. Ethology. 2022;128(3):275–84. doi: 10.1111/eth.13251

[62] Hobson EA, Avery ML, Wright TF. An analytical framework for quantifying and testing patterns of temporal dynamics in social networks. Anim Behav. 2013;85(1):83–96. doi: 10.1016/j.anbehav.2012.10.010

[63] Smit N. Hierarchies inferred from different agonistic behaviours are not always comparable. J Anim Ecol. 2024;93(12):1947–59. doi: 10.1111/1365-2656.14203 39435973 PMC11615268

[64] R Core Team. R: A language and environment for statistical computing. R Foundation for Statistical Computing [Internet]. Vienna, Austria: R Foundation for Statistical Computing; 2025. Available from: https://www.r-project.org/

[65] Wickham H. ggplot2: Elegant Graphics for Data Analysis [Internet]. New York: Springer-Verlag; 2016. Available from: https://ggplot2.tidyverse.org

[66] Soetaert K. diagram: Functions for visualising simple graphs (networks), plotting flow diagrams [Internet]. R package version 1.6.5. 2020. Available from: https://cran.r-project.org/package=diagram

[67] Wilke CO. ggridges: Ridgeline Plots in ‘ggplot2’ [Internet]. R package version 0.5.3. 2021. Available from: https://cran.r-project.org/package=ggridges

[68] Brooks ME, Kristensen K, Benthem KJ van, Magnusson A, Berg CW, Nielsen A. glmmTMB balances speed and flexibility among packages for zero-inflated generalized linear mixed modeling. R J. 2017;9(2):378–400.

[69] Lüdecke D, Ben-Shachar M, Patil I, Waggoner P, Makowski D. performance: An R Package for Assessment, Comparison and Testing of Statistical Models. JOSS. 2021;6(60):3139. doi: 10.21105/joss.03139

[70] Hartig F. Residual diagnostics for hierarchical (multi-level/mixed) regression models. R package ver 302. 2017.

[71] Koller M. Robustlmm: An R package for robust estimation of linear mixed-effects models. J Stat Softw. 2016;75:1–24. doi: 10.18637/jss.v075.i06 PMC7351245 32655332

[72] Bates D, Mächler M, Bolker B, Walker S. Fitting linear mixed-effects models using lme4. J Stat Softw. 2015;67(1). doi: 10.18637/jss.v067.i01

[73] Zeileis A. Diagnostic checking in regression relationships. R News. 2002;2(3):7–10.

[74] van der Marel A, Hobson EA. Monk_dompattern: Code and date for social perturbation experiment of dominance patterns in monk parakeets [Internet]. annemarievdmarel/Monk_dompattern: GitHub. 2022. Available from: https://github.com/annemarievdmarel/Monk_dompattern

