# Supplementary material for: Social manipulations trigger shifts in group-level dominance patterns
Source: PLoS Comput Biol. 2026 Nov 5;22(11):e1014875. doi: 10.1371/journal.pcbi.1014875 (PMC13739146; doi:10.1371/journal.pcbi.1014875)
Supplement: Supplementary file 1 [file pcbi.1014875.s001.docx]

# S1 Appendix

**Social manipulations trigger shifts in group-level dominance patterns**

Annemarie van der Marel^1,2,*^, Xavier Francis^1^, Claire L. O’Connell^1,3^, Cesar O. Estien^1,4,5^, Chelsea Carminito^1^, V. Darby Moore^1^, Elizabeth A. Hobson^1^

*** [avdmarel@outlook.com](mailto:avdmarel@outlook.com)

^1^ Department of Biological Sciences, University of Cincinnati, Cincinnati, OH, United States of America.

^2^ Current address: Instituto de Ecología y Biodiversidad (IEB), Santiago, Chile.

^3^ Department of Biological Sciences, Thomas More University, Crestview Hills, KY, United States of America.

^4^ Department of Environmental Science, Policy, and Management, University of California–Berkeley, Berkeley, CA, United States of America.

^5^ Second Nature Ecology & Design LLC, United States of America.

**List of Tables**

[**Table A.** The schedule of the experimental setup to study dominance patterns outside of experimental manipulations in monk parakeets.	3](#_Toc242285531)

[**Table B.** The 2021 experimental social perturbation schedule to study the effects of the perturbation of a key individual in monk parakeets.	4](#_Toc242285532)

[**Table C.** The 2022 experimental social perturbation schedule to study the response to the perturbations of differently ranked birds in monk parakeets..	5](#_Toc242285533)

[**Table D.** Sensitivity analysis of aggression balance**.**	8](#_Toc242285534)

**List of Figures**

[**Figure A.** A conceptual figure of the analysis to test how unusual the observed pattern changes are after top-ranked perturbations**.**	7](#_Toc242285417)

[**Figure B.** The monk parakeet aggression networks are consistent throughout the social experiment in 2021.	8](#_Toc242285418)

[**Figure C.** Aggression balance over time**.**	9](#_Toc242285419)

[**Figure D.** Number of aggressors of focal birds and similar ranked birds.	10](#_Toc242285420)

[**Figure E.** Focus and position to categorize dominance patterns.	11](#_Toc242285421)

[**Figure F.** Dominance patterns and targeted aggression both changed differently depending on the rank of the perturbed individual and perturbation type..	12](#_Toc242285422)

[**Figure G.** Summary of the number of pattern transitions across all trials and experimental social groups.	13](#_Toc242285423)

**Table A.** The schedule of the experimental setup to study dominance patterns outside of experimental manipulations in monk parakeets.

| Start date | End date | Rank assessment period | Days | Hours observed | Birds | Agonistic events | # dyads |
| --- | --- | --- | --- | --- | --- | --- | --- |
| ***2020 social group*** | | | | |  |  |  |
| 2020-03-18 | 2020-03-20 | Bin 1 | 3 | 16.5 | 21 | 784 | 159 |
| 2020-03-21 |  | Capture 1 | 1 |  |  |  |  |
| 2020-03-22 | 2020-03-24 | Bin 2 | 3 | 21.5 | 21 | 1161 | 181 |
| 2020-05-25 | 2020-05-26 | days off | 2 |  |  |  |  |
| 2020-03-27 | 2020-03-29 | Bin 3 | 3 | 23.4 | 21 | 1203 | 203 |
| 2020-03-30 |  | Capture 2 | 1 |  |  |  |  |
|  | total |  | 13 | 61.4 |  | 3148 |  |
| ***2021 social group*** | |  |  |  |  |  |  |
| 2021-04-06 | 2021-04-08 | Bin 1 | 3 |  | 22 | 1855 | 304 |
| 2021-04-09 | 2021-04-10 | Days off | 2 |  |  |  |  |
| 2021-04-11 |  | Thunderstorm at night |  |  |  |  |  |
| 2021-04-11 | 2021-04-13 | Bin 2 | 3 |  | 22 | 1063 | 286 |
| 2021-04-14 | 2021-04-16 | Bin 3 | 3 |  | 22 | 1572 | 292 |
| 2021-04-17 | 2021-04-19 | Bin 4 | 3 |  | 22 | 1329 | 245 |
| 2021-04-20 | 2021-04-22 | Bin 5 | 3 |  | 22 | 1560 | 262 |
| 2021-04-23 |  | Day off | 1 |  |  |  |  |
| 2021-04-24 | 2021-04-26 | Bin 6 | 3 |  | 22 | 2439 | 316 |
| 2021-04-27 |  | Capture | 1 |  |  |  |  |
| 2021-04-28 | 2021-05-01 | Bin 7 | 4 |  | 20 | 2417 | 265 |
| 2021-05-02 | 2021-05-03 | Day off | 2 |  |  |  |  |
| 2021-05-04 | 2021-05-06 | Bin 8; partner switch | 3 |  | 20 | 2437 | 266 |
| 2021-05-07 | 2021-05-09 | Bin 9 | 3 |  | 20 | 2203 | 262 |
| 2021-05-10 | 2021-05-12 | Bin 10 | 3 |  | 20 | 1983 | 236 |
| 2021-05-13 | 2021-05-14 | Day off | 2 |  | 20 |  |  |
|  | TOTAL |  |  |  |  | 18858 |  |

**Table B.** The 2021 experimental social perturbation schedule to study the effects of the perturbation of a key individual in monk parakeets. In total, we performed three perturbation trials, which lasted 17 days with an 8-day removal and an 8-day reintroduction period. We pooled three days to assess dominance patterns rendering 12 dominance pattern assessment periods.

| Start date | End date | Trial | Rank assessment period | Days | Hours observed | Birds | Agonistic events | # dyads |
| --- | --- | --- | --- | --- | --- | --- | --- | --- |
| 2021-04-05 | 2021-05-15 | na | Initial group formation | | |  |  |  |
|  |  |  |  |  |  |  |  |  |
| 2021-05-16 | 2021-05-18 | 1 | Bin 11 | 3 | 22.85 | 20 | 1977 | 242 |
| 2021-05-19 |  | 1 | capture 1 | 1 |  |  |  |  |
| 2021-05-20 | 2021-05-22 | 1 | Bin 12 | 3 | 21.82 | 19 | 2111 | 227 |
| 2021-05-23 | 2021-05-24 | 1 | days off | 2 |  |  |  |  |
| 2021-05-25 | 2021-05-27 | 1 | Bin 13 | 3 | 22.58 | 19 | 1738 | 226 |
| 2021-05-28 | 2021-05-30 | 1 | Bin 14 | 3 | 26.7 | 20 | 2928 | 263 |
| 2021-05-31 | 2021-06-01 | 1 | days off | 2 |  |  |  |  |
| 2021-06-02 | 2021-06-04 | 2 | Bin 15 | 3 | 21.38 | 20 | 1856 | 235 |
| 2021-06-05 |  | 2 | capture 2 | 1 |  |  |  |  |
| 2021-06-06 | 2021-06-08 | 2 | Bin 16 | 3 | 20.95 | 19 | 2218 | 213 |
| 2021-06-09 | 2021-06-10 | 2 | days off | 2 |  |  |  |  |
| 2021-06-11 | 2021-06-13 | 2 | Bin 17 | 3 | 19.68 | 19 | 1887 | 220 |
| 2021-06-14 | 2021-06-16 | 2 | Bin 18 | 3 | 18.37 | 20 | 1713 | 218 |
| 2021-06-17 | 2021-06-18 | 2 | days off | 2 |  |  |  |  |
| 2021-06-19 | 2021-06-22 | 3 | Bin 19 | 3.5 | 20.97 | 20 | 1615 | 224 |
| 2021-06-23 |  | 3 | capture 3 | 1 |  |  |  |  |
| 2021-06-24 | 2021-06-26 | 3 | Bin 20 | 3 | 17.82 | 19 | 897 | 178 |
| 2021-06-27 | 2021-06-28 | 3 | days off | 2 |  |  |  |  |
| 2021-06-29 | 2021-07-01 | 3 | Bin 21 | 3 | 21.38 | 19 | 2131 | 224 |
| 2021-07-02 | 2021-07-04 | 3 | Bin 22 | 3 | 20.98 | 20 | 2005 | 229 |
|  | TOTAL |  |  | 40 | 255.5 |  | 23076 |  |

**Table C.** The 2022 experimental social perturbation schedule to study the response to the perturbations of differently ranked birds in monk parakeets. In total, we performed six perturbation trials, which lasted 17 days with an 8-day removal and an 8-day reintroduction period. We pooled three days to assess dominance patterns rendering 26 dominance pattern assessment periods.

| Start date | Trial | Rank assessment period | Hours observed | Birds | Agonistic events | |
| --- | --- | --- | --- | --- | --- | --- |
|  |  |  |  |  | Group 1 | Group 2 |
| 2022-01-19 |  | 1 | 35.05 | 11 | 2175 | 2050 |
| 2022-01-26 | 1 | 2 | 16.07 | 11 | 1725 | 1692 |
| 2022-01-30 | 1 | 3 | 11.70 | 10 | 1245 | 1715 |
| 2022-02-04 | 1 | 4 | 18.83 | 10 | 1068 | 1071 |
| 2022-02-07 | 1 | 5 | 17.90 | 11 | 1500 | 2507 |
| 2022-02-12 | 2 | 6 | 18.48 | 11 | 3142 | 2080 |
| 2022-02-16 | 2 | 7 | 18.78 | 10 | 1458 | 1459 |
| 2022-02-21 | 2 | 8 | 19.20 | 10 | 1505 | 1395 |
| 2022-02-24 | 2 | 9 | 19.22 | 11 | 1817 | 1374 |
| 2022-03-01 | 3 | 10 | 19.55 | 11 | 1472 | 1182 |
| 2022-03-05 | 3 | 11 | 19.40 | 10 | 1171 | 1031 |
| 2022-03-10 | 3 | 12 | 9.77 | 10 | 806 | 582 |
| 2022-03-14 | 3 | 13 | 14.45 | 11 | 1503 | 1709 |
| 2022-03-19 | 4 | 14 | 17.07 | 11 | 1067 | 1294 |
| 2022-03-23 | 4 | 15 | 19.23 | 10 | 2098 | 1714 |
| 2022-03-28 | 4 | 16 | 20.37 | 10 | 1899 | 1105 |
| 2022-03-31 | 4 | 17 | 19.68 | 11 | 3366 | 2066 |
| 2022-04-05 | 5 | 18 | 19.00 | 11 | 2595 | 1676 |
| 2022-04-09 | 5 | 19 | 16.70 | 10 | 1623 | 1025 |
| 2022-04-14 | 5 | 20 | 17.72 | 10 | 1668 | 1380 |
| 2022-04-17 | 5 | 21 | 19.98 | 11 | 1328 | 1600 |
| 2022-04-22 | 6 | 22 | 20.30 | 11 | 924 | 1665 |
| 2022-04-26 | 6 | 23 | 19.45 | 10 | 1370 | 2254 |
| 2022-05-01 | 6 | 24 | 21.28 | 10 | 1373 | 2060 |
| 2022-05-04 | 6 | 25 | 18.90 | 11 | 1443 | 2985 |
| 2022-05-09 |  | 26 | 13.25 | 11 | 939 | 1731 |
|  |  | TOTAL |  |  | 42280 | 42403 |
|  |  |  |  |  |  |  |

**Fig A.** **A conceptual figure of the analysis to test how unusual the observed pattern changes are after top-ranked perturbations.** We created a reference model that randomized the observed social dominance patterns for 1000 iterations (Step 1). We compared the social dominance patterns for the 3-day period immediately before the perturbation (removal or reintroduction) to the 3-day period immediately after the perturbation. Using these 1000 random pattern change distributions; we calculated the transition probabilities across all 1000 runs (Step 2A) and the proportion of pattern changes out of 1000 runs (Step 2B). Here we highlighted the randomisation by perturbation type of only the top-ranked perturbation trials.

**Fig B.** **The monk parakeet aggression networks are consistent throughout the social experiment in 2021.** Panel (a) shows the aggression networks for the 3-day periods before and after removal and panel (b) the networks for the periods before and after reintroduction. The nodes are ordered by their respective power score with higher ranking birds closer to 1. For the periods before removal and after reintroduction, the focal birds are highlighted with different colors for each trial. The edge width represents the total number of agonistic events between two individuals. The blue edges represent the rule-followers (higher-ranking birds aggress against lower-ranking birds, mean ± SD = 59.6 % ± 4.5 %), and the red edges represent the rule-breakers (lower-ranking birds aggress against higher-ranking birds, mean ± SD = 40.5 ± 4.5 %). The period numbers are the social dominance pattern assessment periods starting from the experimental period.

**Fig C.** **Aggression balance over time.** Each panel represents different social groups. Values of 1 represent birds that only aggress, while values closer to 0 means that birds mainly received aggression. Focal birds are highlighted.

**Table D.** **Sensitivity analysis of aggression balance.** Results of the binomial generalized linear mixed model of aggression counts (aggression given vs received) with logit link. Shown are odds ratios for aggression after reintroduction relative to before removal, by focal rank and focal status. Random intercepts for bird ID and group were included.

| Focal rank | Focal status | Odds ratio (after / before) | SE | z | p-value |
| --- | --- | --- | --- | --- | --- |
| lower‑ranked | focal | 0.27 | 0.0276 | −12.82 | < 0.0001 |
| top‑ranked | focal | 0.0008 | 0.00018 | −32.50 | < 0.0001 |
| lower‑ranked | nonfocal | 1.23 | 0.0271 | 9.30 | < 0.0001 |
| top‑ranked | nonfocal | 1.72 | 0.0308 | 30.00 | < 0.0001 |

**Fig D.** **Number of aggressors of focal birds and similar ranked birds**. The total number of distinct aggressors controlled for by group size was the same for the focal birds upon reintroduction compared to similarly ranked birds prior to the focal’s reintroduction (LR test: λ = 2.85, P = 0.24). Number of distinct aggressors controlled for by group size were the same when a top-ranked or a middle/low-ranked bird was perturbed (LR test: λ = 0.36, P = 0.84). We found no interaction effect for number of distinct aggressors between the rank of the focal birds and the subject (LR test: λ = 0.35, P = 0.56).

**Fig E. Focus and position to categorize dominance patterns.** Panel a) shows a visual example (period 1 group 2021) of how dominance patterns are categorized based on the reference model and the polygon around the 95% confidence intervals of the tuning parameter. Panel b) visualizes focus and position values for all 3-day periods and all social groups. This figure highlights that distinct social groups can have similar focus and position values which can lead to different social dominance patterns due to underlying aggression structure.

**Fig F.** **Dominance patterns and targeted aggression both changed differently depending on the rank of the perturbed individual and perturbation type**. Using transition diagrams, we summarized dominance patterns transitions for both groups in 2022 after (a) removals and (b) reintroductions of (i) top-ranked and (ii) middle/low-ranked focal birds.

**Fig G.** **Summary of the number of pattern transitions across all trials and experimental social groups.** W we summarized the dominance pattern transitions across all trials and social groups. The monk parakeet groups showed pattern transitions in 53.3% of the perturbation events, where removals caused 46.7% and reintroductions caused 60.0% shifts in pattern use. Top-ranked perturbations caused 66.7% pattern shifts, while middle/low-ranked perturbations caused 33.3% shifts. Top-ranked perturbations resulted in more social upheaval, therefore, we focus on these trials to study how top-ranked perturbations affected the dominance patterns dynamics. Panels show pattern transitions for each perturbation type: (a) both removal and reintroduction perturbations, (b) just removal perturbations, and (c) just reintroduction perturbations. Within each panel, the bars show percent of pattern transitions observed for each rank treatment: black shows the combined outcomes, orange shows only the top-ranked perturbations, and light grey shows only the mid/low-ranked perturbations. The number of transitions is labeled in bold above each bar and the number of perturbations of each type is labeled at the base of each bar (text in white boxes).
