# Supplementary material for: Social manipulations trigger shifts in group-level dominance patterns
Source: PLoS Comput Biol. 2026 Nov 5;22(11):e1014875. doi: 10.1371/journal.pcbi.1014875 (PMC13739146; doi:10.1371/journal.pcbi.1014875)
Supplement: Supplementary file 2 [file pcbi.1014875.s003.docx]

PCOMPBIOL-D-26-00608
Social manipulations trigger shifts in group-level dominance patterns
PLOS Computational Biology


**Thank you for the opportunity to revise our manuscript. We are happy to see that the reviewers were positive about the question, system, and methods. Both reviewers did have several areas where they wanted more clarification, details, and justification, which were valuable suggestions and helped us significantly improve the manuscript. We addressed these comments in detail below in our response letter, but generally:**

(1) **We added a definitions table to clarify our term usage to avoid confusion and improve consistency,**
(2) **We included more details in the introduction and discussion**
(3) **We updated Figure 2 and the analysis on change in aggression balance instead of absolute change in aggression balance.**
(4) **We added analyses to our question of the effects of dominance rank on number of pattern shifts and the use of patterns.**
(5) **We also addressed concerns regarding our experimental setup.**

**We believe our current version addresses all the editor’s and reviewer’s concerns. The analyses that we added based on the reviewer suggestions enriched the paper, but did not change our original results or interpretation of the results.**

**Page and line numbers correspond to the revised manuscript without the track changes.**

**Journal Requirements:**

1) Please provide an Author Summary. This should appear in your manuscript between the Abstract (if applicable) and the Introduction, and should be 150-200 words long. The aim should be to make your findings accessible to a wide audience that includes both scientists and non-scientists. Sample summaries can be found on our website under Submission Guidelines:
<https://journals.plos.org/ploscompbiol/s/submission-guidelines#loc-parts-of-a-submission>

2) We do not publish any copyright or trademark symbols that usually accompany proprietary names, eg ©,  ®, or TM  (e.g. next to drug or reagent names). Therefore please remove all instances of trademark/copyright symbols throughout the text, including:
- ® on page: 20.

3) Please upload all main figures as separate Figure files in .tif or .eps format. For more information about how to convert and format your figure files please see our guidelines: 
<https://journals.plos.org/ploscompbiol/s/figures>

4) We have noticed that you have uploaded Supporting Information files, but you have not included a list of legends. Please add a full list of legends for your Supporting Information files after the references list.

5) Please ensure that the funders and grant numbers match between the Financial Disclosure field and the Funding Information tab in your submission form. Note that the funders must be provided in the same order in both places as well.

Note: If the reviewer comments include a recommendation to cite specific previously published works, please review and evaluate these publications to determine whether they are relevant and should be cited. There is no requirement to cite these works unless the editor has indicated otherwise.

**Changed, included, and updated these points accordingly.**

# Reviewer 1

Reviewer #1: I think this is an interesting manuscript presenting some clearly designed experiments, and providing useful insights for the study of dominance and aggression. Yet, I think that the readability can be significantly improved, and certain points are important to be clarified.

**Thank you for your constructive feedback. We agree that readability could be improved and that some points needed clarification. Please find our responses to your concerns below.**

First, I think that the authors can improve the consistency of the terminology which currently makes the text hard to follow (e.g., aggression dynamics vs aggression decisions vs dominance dynamics OR aggression vs agonistic interaction OR dominance rank vs social role vs power score).

**Thank you for the suggestion. This was also a concern of reviewer 2, so we carefully considered which terms to use and used them now consistently. To better and more clearly clarify the terms we used, we added a table with our definitions (see Table 1 in revised version). We also added reminders about term definitions in each section or figure legend.**

**For term consistency: We chose “aggression” over agonism, and “dominance rank” over social role. We kept “power score” as that is how we quantified the dominance rank but more clearly defined power score in the paper. We decided to keep “dominance pattern” as that is the term used for this specific aggression pattern in the previous literature (Hobson et al. 2021) and we wanted to maintain consistency within the literature. We also think keeping the term is justified as it is the recurring way aggression is distributed or directed within the group (the outcome of rank-informed aggression decisions of the group members).**

Second, this article is heavily based on a previous one on dominance patters and aggression heuristics (Hobson et al 2021). I think that the authors need to more thoroughly explain the differences of the dominance patterns in this new article, so the reader can smoothly follow the reasoning. While there are some details in the methods, I think that some of it can be introduced earlier.
**Thank you for highlighting this. It is always difficult to find a balance between including enough information about a recently-published paper in a follow-up study. We followed your suggestion and included more detail on how the dominance patterns were assessed conceptually, but without making it too methods-focused.**

**We now include the text: “Within the context of dominance, these patterns are emergent properties that arise from the consensus of individual aggression decisions across the group, not traits of single individuals. This distinction between individual behavior and group-level emergent patterns is central to understanding social plasticity: while individuals adjust their aggression, the outcome can be a reorganization of the group’s overall social structure. Dominance patterns are identified based on the relationship between the rank of the aggressor and the receiver, comparing how concentrated aggression is on receivers at similar versus different rank distances and where along the dominance hierarchy aggression is most concentrated.” (P3)**

**We also included more details on how the dominance patterns were categorized in the methods section ‘social dominance pattern assessment’. P32-33**

Abstract: I know that we are often supposed to have a fancy 1-2 sentence intro in our abstracts highlighting an important gap of knowledge. I think that the first three sentences of the abstract do not flow well. Mentioning dynamic social systems, social plasticity, hierarchies, aggression etc makes them hard to follow. The authors may want to simplify them. Then, again, in L30 “To investigate factors affecting dominance patterns” starts quite general. The authors could simply write directly sth like “We studied the link…”. I do not see why to refer to “dominance patterns” when one can simply say sth like “rank dependent aggression”, or “aggression heuristics” that has been used in the past
Hobson et al 2021 Aggression heuristics underlie animal dominance hierarchies and provide evidence of group-level social information
Smit & Robbins 2025 Risk-taking incentives predict aggression heuristics in female gorillas
Finally, the last sentence of the abstract, I feel that is again introducing new concepts without fully explaining the relevance. At the end of the discussion it is clearer.

**Thank you for the suggestions. We updated the abstract following your recommendations.**

L47: why “especially in groups structured by aggression and dominance hierarchies “ ?
**Thank you for pointing this out, we agree this phrasing wasn’t as clear as we wanted. We changed especially to “such as”, because social plasticity is also important in other contexts. (L55, P3)**

L49: I think that again this “especially… rather…” needs explanation.
**As above, we agree we could phrase this more clearly. Upon revision, we removed this sentence altogether as using dominance patterns can be cognitive and social history based, but it can also be the outcome of a simple rule like “fight anyone slightly smaller than me” which would also result in a close competitors pattern.**

L51: I find “consensus among group members on who to aggress” unclear. I assume the authors mean depending on rank? I think that using a term like “rank-based aggression” or sth similar would be more clear.

**We rephrased this similar to how we phrased it in the abstract. It now reads as: “When multiple individuals make similar rank-informed aggression decisions, a group-level social dominance pattern can emerge”. (L58, P3)**

L52: I understand “dominance” as something that characterizes relationships, and aggression something that can be instantaneous, characterizing interactions. Thus, I would not use aggression and dominance patterns as equivalent.

**Good point, we removed ‘aggression pattern’.**

L65: Why using “social role (dominance rank)” and not directly “dominance rank”

**We now use dominance rank directly.**

L67: “are highly social parrots” ? (plural)

**Changed accordingly.**

L70: Here you first mention “aggression dynamics” which, I think, is more accurate.

**Upon both your and reviewer 2’s suggestions, we carefully changed the terms used in the paper. We now use the same terms throughout. For example, we now mention in our research aims, whether social perturbations would 1) alter aggression dynamics, and 2) trigger dominance pattern shifts. P4**

L79: So, reintroduction of a previously high ranking individual, would take things back in the previous hierarchy smoothly or the reintroduced individual would occupy a rank at the bottom of the hierarhcy?
**Based on our earlier study, we knew that focal individuals were not able to reoccupy their previous rank immediately on reintroduction (A. van der Marel, *et al.*, Perturbations highlight importance of social history in parakeet rank dynamics. *Behavioral Ecology* 34, 457–467 (2023)). As we already published this, we did not make a separate prediction but included that focal birds did not reoccupy previous rank position upon reintroduction in the introduction. This now reads as follows: “Earlier work with the same social groups and perturbation experiments focused on individual changes in dominance rank and whether rank was determined by individual characteristics or short- or long-term social history, i.e., the memory of social interactions (12). This study found that all focal birds (independent of rank) did not reoccupy their previous rank position upon reintroduction implying that short-term social history (the interactions during the removal period) was the rank determinant over individual characteristics and longer-term social history (12). In the current study, we used data from the same experiments, but focused our analyses here on the group-level response to the perturbations.” (P4)**

**We used these previous results in our discussion, where we state that in this species, that rank appears to be based on social history rather than individual characteristics (P14-15).**

L85: I guess this is an expected comment: why 3 days? I understand that the authors need a threshold, but maybe a little explanation would help.

**Good question, we added more detail about this to the manuscript to better describe and justify our timeline (see limitations section, P16-17). This is also something Reviewer 2 requested.**

**To clarify, we selected 8 days for each removal and reintroduction period as that was short enough so that we were able to perform sufficient trials within a field season but long enough so that the group had an ample chance to restabilize, which was found to be sufficient in earlier studies with monk parakeets in captivity. We kept using the same interval for each field season, so that we could compare replicate trials.**

**In the middle of each removal and reintroduction, the observers had a 2-day break in observations (no data collected, See black vertical line in Figure below). So that is how we selected 3 days. This criterion also resulted in sufficient data per period. See also methods P32**

L87: Does “power score” mean simplify “dominance rank”? If yes, then maybe simply use this term? If not, using the term “power” here for first time, without defining it, is confusing, as it is a ‘parent category’ of dominance (for example: Davidian et al 2022 The eco-evolutionary landscape of power relationships between males and females).

**Thanks for raising this point. "Power score" does not mean exactly the same thing as "dominance rank” as it refers to a continuous rank score (known as “power”) rather than an ordinal rank. We've added a definition to clarify this distinction. The sentence now reads: "...determined each individual's dominance rank using a network-based ranking algorithm based on PageRank that calculated a continuous rank score, called power score." P6**

L91: Here is another new term “agonistic”. Is this the same with “aggressive”? If not explanation of the difference is missing; if yes, why use another term and not being consistent?

**We apologize for the mixed terminology. For clarity, we removed all instances of agonistic in the manuscript and now just use “aggressive” or “aggression”.**

L111: Put all this numbers in a parenthesis at the end to improve readability? (0.26 ± 0.02... vs 0.09…)

**Thank you for the suggestion, but we decided to keep it as is. Otherwise, the sentence would become much longer since we'd also need to add "top-ranked" and "lower-ranked" to the parentheses.**

L114: I am a bit confused here on why the authors compare top-ranked focal birds with group members from middle/low-ranked trials (and middle/low-ranked focal birds with group members from top-ranked trials), instead of comparing focal birds of different rank as well as remaining group memebers of different rank.
**The interaction shows the aggression balance for focal birds in top-ranked trials or lower-ranked trials and group members in top-ranked and lower-ranked trials. This was in addition to testing for the difference in aggression balance for all trials (both top- and lower-ranked) between focal birds and remaining group members and the difference among all birds between top- and lower-ranked trials.**

**We now clarified this in the ‘aggression dynamics’ method section (P33).**

L117: “depending on their” instead of “compared to its”?

**Changed accordingly.**

L123: In almost all text the authors use “middle/low”. I think this is a bit unclear if they refer to two different categories for which the results were always similar, or one category. If the latter, maybe use one word?

**Thank you for the suggestion. As it is one category, we have changed "middle/low-ranked" to "lower-ranked" throughout the manuscript and have now defined the lower-ranked category in the Methods section. P31: “In 2022, we performed trials of three top-ranked, two middle-ranked and one low-ranked bird per group. We combined the latter two into a lower-ranked trial category as comparison to the top-ranked trials.”**

L53: Referring to dominance patterns here made me go back to the first mention of those. In L53 the authors say that “consensus among group members on who to aggress...a dominance pattern can emerge”. But later on, in L129, they report percentages which, I assume, mean that 45% of all aggression was towards “any individual lower-ranked than self”, 33% was “towards much lower-ranked individuals” etc. So, is the dominance pattern a group trait (consensus OR) or an individual trait? Finally, aren’t the close competitors and bullying patterns subcategories of downward heuristic?

**The dominance patterns are group traits. These patterns arise when most group members make the same aggression decisions of where to direct their aggression. We understand where the confusion comes from. In the introduction, we now mention “Within the context of dominance, these patterns are emergent properties that arise from the consensus of individual aggression decisions across the group, not traits of single individuals.” L 61.**

**In the results, we rephrased the sentences to reflect that the patterns are group traits and not that 45% of the individuals aggress toward any individual ranked lower than self. Instead, the percentages are the number of assessment periods the particular pattern was found across the entire study period (77 assessment periods).**

**See P8:** “**The most common pattern was the downward heuristic (attack any individual lower-ranked than self), which was found in 35 out of the 77 assessment periods (45%), followed by the bullying pattern (preferentially attack much lower-ranked individuals) in 25 out of 77 periods (32%), and the close competitors pattern (attack slightly lower-ranked individuals) in 17 out of 77 periods (22%; Table 3).”**

L179-182: Some of 1,2,3 include methods and other results.

**Changed accordingly.**

L187: If it is “the history of social interactions”, why the re-introduced individuals don’t reoccupy they previous positions smoothly? Seems like there is a temporal aspect. You somehow discuss that later on, but maybe the “history of insteraction” is not the actual determinant?
**Thank you for this comment. You are right that there is a temporal aspect here. Precisely because of the short-term history of social interactions, the focal bird does not reoccupy its previous position smoothly as the remaining group members have updated their social information during the focal bird's removal.**

**In contrast, if it was based on longer-term history (interactions prior to removal) or individual characteristics, such as size, then it could have returned to the hierarchy at the same position. So, rank is more based on recent social interactions than long-term history because removal creates a gap in interaction history, so the bird’s “social memory” in the group is weakened. Social history may be dynamic, with older interactions decaying in influence with perturbations resetting that history. (A. van der Marel, *et al.*, Perturbations highlight importance of social history in parakeet rank dynamics. *Behavioral Ecology* 34, 457–467 (2023).)**

**We now include the results of this earlier work in the introduction to better highlight this process:**

**“Earlier work with the same social groups and perturbation experiments focused on individual changes in dominance rank and whether rank was determined by individual characteristics or short- or long-term social history, i.e., the memory of social interactions. This study found that all focal birds (independent of rank) did not reoccupy their previous rank position upon reintroduction implying that short-term social history (the interactions during the removal period) was the rank determinant over individual characteristics and longer-term social history (10). In the current study, we investigate the group-level response to the perturbations.” P4**

L196: I do not understand the “shifts within the same group from multiple groups”
**We rephrased this for clarity. The sentence now reads as: “We documented 29 shifts in dominance patterns, with different groups showing multiple shifts over time” P13.**

L220: Some other relevant refs to the point that I found. I am not asking the authors to cite this work. I just found these articles quite relevant.
Franz et al 2015 Knockouts of high-ranking males have limited impact on baboon social networks
Ewbank & Meese 2010 Aggressive behaviour in groups of domesticated pigs on removal and return of individuals
Piefke et al 2021 Social network stability is impacted by removing a dominant male in replicate dominance hierarchies of a cichlid fish

**Thank you for the suggestions. We added the Piefke citation on P15, as we mention here other species where perturbations of top-ranked individuals lead to social structure changes in the group. We did not include Ewbank & Meese as they found the opposite, where lower-ranked focals had a harder time than top-ranked focals. Franz also found limited effect on social structure changes in the baboon networks.**

L232: I am wondering if a shift in dominance pattern necessarily means “social destabilization”.
**You are correct, a shift does not necessarily mean social destabilization. We removed this statement.**

L380: If I understand correctly, aggression balance is an individual property, while you start the paragraph referring to “each group and pattern assessment period”

**That is correct. We removed ‘each group’ for clarity.**

I admit that I didn’t manage to focus well on the methods, because of the inconsistencies in concepts etc I highlighted above. However, I think that the methods had generally a better flow.

**Thank you for your help!**

# Reviewer 2

Reviewer #2: This paper entitled “Social manipulations trigger shifts in group-level dominance patterns” addresses changes in dominance patterns following experimental removals or reintroductions of individuals of different ranks. I quite appreciate the depth of detail in the manuscript and there is a great wealth of analyses. I have highlighted below some of my concerns. I think the paper would greatly benefit from an overall increase of sharpness. I find it at times quite hard to read and follow, there are a lot of different terms and a bit of jargon that makes the reader continuously have to go back and forth for definitions. I think that addressing some of the concerns I raise in this review could really help. I hope that I can make it clear why I have come to this conclusion, and that the authors can address these in the revisions (or better explain their work if I have misunderstood).

Samuele Ramellini
(I sign all of my reviews)

**Thank you for your constructive feedback. The comments were clear and we either addressed the comments or explained ourselves better in this revision.**

Relevance: My main question is related to why this is relevant in nature. While I appreciate the mention of fission fusion dynamics, I was honestly expecting much more discussion about how these mechanisms have emerged and how the specific social system of the species might have favoured/fixed these mechanisms. The authors seem to suggest that group composition should be robust to changes. However, it seems that dominance is changing all the time, because of perturbations but also just randomly. How does this tie in with the social system of the species? How could this be extended to other species with the same social structure? The last few sentences seem to be pointing a little bit in this direction, but these are just left at the end of the manuscript, not really introduced or discussed anywhere else.
**Thank you for raising this question about ecological relevance. For several reasons, we did not translate our results to the social system of the species or species with similar social systems.**

**The reason for us to conduct these social manipulation experiments in captivity is that this experimental approach allowed us to determine what the species is capable of in terms of social responses. Particularly, as it is challenging to establish causality in wild populations, where so many other factors could affect group’ responses that you cannot control.**

**Furthermore, monk parakeets are difficult to study in the wild, particularly regarding social structure. For example, it is not yet known whether they maintain dominance hierarchies in natural populations. Their primary social unit is a pair; they nest colonially in large stick nests and flock with other social units during flight or foraging. These fission–fusion dynamics occur on a daily basis and are similar to fission-fusion dynamics found in other parrot species. Where the monk parakeets differ is in that they are social all year-round in contrast to most parrot species that live in breeding pairs during the breeding season.**

**Given the limited information available about this species in the wild, we focused our interpretation on what the species is capable of rather than speculating about how our findings may apply to wild populations. Our key finding is that monk parakeets show social plasticity and respond differently across distinct contexts.**

**The next step would be to study this species in the wild and perform comparative studies to understand how these mechanisms operate under natural conditions. We have included this in the new limitations section in the discussion. (see P16)**

Focus of introduction: I found the intro is very based on individual level changes while the rest of the paper actually focuses strongly on group level dominance pattern changes. Maybe referring to “emergent” patterns and explore the literature on this a little bit more would ease the transition and help the reader framing the results.

**Thank you for the suggestion. We updated our second intro paragraph and included more info on emergent patterns. We added: “Emergent group-level patterns are well-documented, where simple individual rules result in collective behavior and give rise to complex group-level structures, such as collective movement or synchronized vigilance (9–11). Within the context of dominance, these patterns are emergent properties that arise from the consensus of individual aggression decisions across the group, not traits of single individuals. This distinction between individual behavior and group-level emergent patterns is central to understanding social plasticity: while individuals adjust their aggression, the outcome can be a reorganization of the group’s overall social structure.” L58-66, P3.**

Perturbation & other definitions: I find the combination of removals and reintroductions into “perturbations” very confusing. I find hard to understand what the predictions are referring to (e.g. L70-L71) and these could be made into sharper and directional predictions for either of the removals or reintroductions.
Rule-following is mentioned only in Fig 2, and never really defined in the text. These are also previously referred as downward heuristics (L53).
There are a lot of terms (e.g. focal individual, rule following, dominance patterns) that are briefly defined but used extensively. A lot of these terms can be redefined or restated as that would really help in following the language across the manuscript.
**Upon both your and reviewer 1’s suggestions, we carefully changed the terms used in the paper. We now use the same terms throughout and included a glossary table (Table 1). We also defined all our terms in the text and redefined the terms in each section.**

**We decided to keep using perturbation to mean both rank-based removals and reintroductions, but we now clearly define this in the text (L104-105) and include the term in the glossary table.**

Interval experiments: The interval between perturbations seems to be really short. For example, there are only a few days between the reintroduction of an individual and the removal of another one (I’m referring to the interval between the 17-days trials). In Fig. 1c, it seems that after the first reintroduction of the top-ranked individuals, there is an increase in the aggression rate. But after one three-day assessment period, the middle ranked are excluded, so it’s not clear if the dynamics went back to being comparable to pre-perturbation levels.

**This was a comment from both reviewer 1 and yours, suggesting that we did not clearly describe our experimental schedule. To be more clear about our experimental timeline, we updated our methods section on rank-based removals (P31).**

**In brief, for each perturbation trial that lasted 17-days, we had four 3-day periods of data collection, except for ‘post-reintroduction’ which was 2 days. In the middle of each removal and reintroduction, the observers had a 2-day break in observations (no data collected). We selected 8 days as that was short enough so that we were able to perform sufficient trials within a field season but long enough so that the group had ample time to restabilize, which was found to be sufficient in earlier studies with monk parakeets in captivity. We now discuss this choice in more detail in our discussion section on potential limitations.**

Finally, how biologically relevant is this interval? Is there any evidence in nature that these events are this regular and this intense (one removal/reintroduction every two weeks)?
**Thank you for raising this concern about biological relevance. We agree that our chosen experimental interval (one removal/reintroduction every two weeks) may not directly mirror natural conditions. Unfortunately, we do not have data on the frequency and intensity of such events in natural monk parakeet groups, which is why we have avoided speculating about this in the paper. However, fission-fusion dynamics occur on a daily basis in this species, suggesting that removals and reintroductions could happen much more frequently in the wild, though these perturbations may involve multiple individuals rather than single birds. Furthermore, demographic processes (such as mortality or dispersal) may occur less frequently, but this would depend on population demographics, predation pressure, and other environmental factors that vary across contexts.**

**In the discussion, we now describe the biological relevance of group membership changes in this species, and describe how it could be an exciting direction for future research. (P16,17)**

Stability of aggression rate: In L99, I agree that this rate is clearly higher, but it seems that this is the baseline level for that group (the pre-perturbation levels in the first two assessments are exactly on the average). It seems that these two groups had just more aggressive background levels. How does this affect the results? How are the four groups compared in this sense?
**The reviewer is correct that some groups had higher background aggression. In the revised version we now updated the methods to account for background aggression. Particularly, we compared each subsequent period against the pre-perturbation baseline by fitting time as a factor and using the baseline period as the reference level. This allowed us to test whether aggression in each later period differed from baseline. We also adjusted this analysis by testing this on number of interactions instead of aggression rate and controlled for group size and hours observed within the model. Results P7, Methods: P33.**

Minor comments

L34. I would consider reversing the order of 1 and 2. It seems like the more important result is the second.
**Thank you for the suggestion. We changed the order. L31**

L36-37. Worth specifying how these reintroductions affected group-level hierarchical organisation.

**Thank you for the suggestion. We now have “In particular, reintroductions of top-ranked birds led to marked changes in aggression and shifts in dominance patterns.” L33.**

L65-67. It’s a bit confusing referring to “individuals” in these sentences. In the first “individual” of L65, I guess perturbation is referring to the removed individual (in which case I would just call it removal). While in the second case, the change in “individual aggression decision-making” is referring to the remaining individuals (the not removed). Maybe restructuring this sentence to reflect these specifics would help solving the confusion. Also, maybe worth going back to the “focal” language.

**Thank you for the suggestion, we restructured the sentence. It now reads as: we tested whether removals and reintroductions of focal individuals differing in dominance rank would 1) change aggression decision-making in the remaining group members and 2) trigger dominance pattern shifts”. P4**

L73. I’m not quite sure what “possible drivers” mean here.
**We changed the sentence. It now reads as: Second, we hypothesized that dominance rank (top-ranked or middle/low-ranked) and removals and reintroductions would induce changes in aggression dynamics and dominance patterns. P5**

L74. What do the authors mean with more changes? More in terms of number, in terms of intensity of changes? Please clarify.
**We clarified this accordingly. “We predicted that top-ranked trials and removals would lead to higher aggression rates, greater aggression balance differences, more shifts in dominance patterns, and different dominance pattern shifts compared to lower-ranked trials and reintroductions, respectively.” P5**

L79. This last prediction is a bit confusing. Is it predicting that the dominance pattern would return to the pre-removal level?

**We updated the last prediction to also include the aggression dynamics and that dominance pattern would remain the same after reintroductions.**

**“We predicted that removals would have a greater impact because the sudden absence of a key individual forces the remaining members to update their ranks. In contrast, after reintroduction, most of the group would have adjusted their ranks, except for the returning bird. Because reintroduced individuals are unlikely to regain their previous rank (12), we expected that the remaining birds would not change their aggression decisions and that dominance patterns would remain stable.” P5**

L81, is the “their” referring to the reintroduced bird or the remaining individuals?

**We rephrased this section to make it more clear we were referring to the remaining birds. P5**

L86-86: Are there events that include more than one individual? Is there any reason to specify that the authors only use directional aggression in L86? Is there any non-directional aggression?

**Yes, there were instances where many birds were involved in aggression, which we called a brawl. These events were too difficult to obtain observations where we could identify winners and losers with confidence. Therefore, we did not include these events. There also was a non-directional aggressive behavior, called spar, where two birds come together with beaks open but ends in a draw.**

Also, in Fig 2a, why is there only one focal individual highlighted? Shouldn’t it be three?

**Apologies for the confusion, we meant to have 2a as an exemplar, rather than showing all the data. We added clarification about this to the revised figure legend.**

L91. This seems a bit repetitive from the previous paragraph.
**As per your suggestion, we removed this sentence.**

L94. I guess this refers to the removal of one top-ranked bird per group as compared to 3 top-ranked birds in 2022.

**Apologies for the confusion. In both groups, we performed 3 perturbation trials, where we removed and reintroduced a top-ranked bird per trial. In 2022, we additionally performed 3 trials where we removed and reintroduced middle-/low-ranked birds. We clarified this in the revised methods (P31): “In our 2021 experiment, we performed removal/reintroduction trials of three top-ranked birds (termed top-ranked trials). In 2022, we performed trials of three top-ranked, two middle-ranked and one low-ranked bird per group. We combined the latter two into a lower-ranked trial category as comparison to the top-ranked trials. We randomized the order of the trials prior to the start of the field season.”**

L101: I’m wondering whether instead of the absolute change in aggression balance, it’d be worth showing the change in general. Are these birds experiencing more or less aggression?
**Upon both your suggestion and the suggestion of reviewer 3, we changed the analysis to the change in general instead of the absolute change. In the first version, we used absolute aggression balance as the distribution was easier to implement (beta distribution) in contrast to robust mixed modeling. In the revised version, we now show the results based on the change in aggression balance (lines 139-147).**

L219: this sentence seems to suggest group coordination in changes. I would refer to these as emergent patterns rather than group shifts. There is not much testing of group decision making in this manuscript.

**Upon revision of our discussion, we removed this sentence.**

# Reviewer 3

Reviewer #3: I liked the system, the main questions and the experimental paradigm of social perturbations involving removal and reintroductions of high-ranked vs lower-ranked individuals. I particularly liked the analyses examining transition probabilities across three types of rank-based aggression patterns and how these transitions might differ for perturbations of top-ranked vs middle/low ranked individuals (versus control situations with no perturbations) and for removals vs reintroductions.

**Thank you.**

I worry that the experimental design had no controls for a removal, all birds were captured and then re-released except for the birds that were ‘removal’ birds. A control would be to capture them all, and then re-release them all. It seems very conceivable that the act of capturing and re-releasing birds causes changes in dominance and aggression patterns. Differences between effects of temporarily removing high ranked vs lower ranked birds could be due to differing effects of the capture/release perturbation per se on high ranked birds (in the treatment that removed lower ranked birds) vs capture/release per se on lower ranked birds (in the treatment that removed higher ranked birds). Differences between removals vs reintroductions could obviously be strongly influenced by the fact that the reintroductions do not involve capture and release of all other individuals. This strikes me as an important flaw in the experimental design that, at minimum, should be discussed head-on, or in a devil’s advocate view, significantly reduces confidence in results.
**Thank you for raising this important point. We agree that capture and release could influence dominance interactions, and we considered this carefully during the study design. We chose not to include an additional “capture/release” control for several reasons.**

**First, each trapping event is stressful for the birds, and minimizing captures was a key welfare consideration. For this reason, we did not include the removal-day data in our analyses (to allow captured birds time to recover from trapping and handling) and limited reintroductions to the focal individual rather than recapturing the entire group.**

**Second, we experienced several unplanned disturbances during the 2021 field season (e.g., a severe storm, injury-related removals, and a dominant partner switch) that disrupted behavior, group composition, and dominance hierarchy, respectively, but none of these perturbations resulted in shifts in dominance patterns. While not a formal control, these events suggest that disturbance alone is not sufficient to produce the effects we found.**

**Finally, logistical constraints also limited the inclusion of additional control treatments, as each trial spanned 17 days and restricted the number of trials per group per season.**

**We now explicitly acknowledge this limitation in the manuscript. We mention the unplanned disturbances (P14) and discuss how capture-related effects could contribute to the observed differences between treatments (P16).**

**This latter part now reads as: “It is important to note that responses to removals and reintroductions should be compared with caveats. On removal days, all birds were trapped and handled, but on reintroduction days, only the reintroduced bird was handled to minimize the number of trapping events for the group. This difference could have influenced how groups responded to removals vs reintroductions. Because of differences in methods, we could not fully separate treatment effects from possible effects of the trapping procedure itself. Since we analyzed aggression excluding the trapping day, the birds should have had sufficient time to recover from handling stress. The patterns we observed can therefore be reasonably, but not certainly, attributed to differential responses to removals and reintroductions rather than just differences in handling.**

Figure 2b shows absolute aggression balance changes (aggression balance upon reintroduction minus aggression balance before removal) for focal birds vs group members, separately for top ranked vs lower ranked birds. While these figures look interesting, to understand what is happening, it would be more useful to see each individual’s balance before removal AND upon reintroduction, with lines joining these values.

**The reviewer raises a good point. For two reasons, we decided against showing the values before and after removals and reintroductions with lines joining the values.**

**First, we performed the analyses on the change of aggression balance calculated as the aggression balance (given/given+received) after minus aggression balance before a perturbation. This change value indicates that an increase in aggression balance from before to after is positive, while decrease is negative. So the change already incorporates in which direction aggression goes for each bird.**

**Second, we visualized the before and after values separately with lines joining but the plots became very busy as we also had many different categories (focalrank, perturbation, subject).**

**To clarify this, we included the following in the methods: “A positive aggression balance indicates an increase in aggression, while a negative value indicates a decrease in aggression from before to after a perturbation.” (P34)**

Ceiling/floor effects (balance scores go from 0 to 1) can obviously affect how much an individual’s balance score could potentially change in one direction or the other, and more importantly, patterns of change (which ones went up versus down) are obviously obscured when one shows the absolute value of changes. Are we really interested in the absolute value of the changes? I would be much more interested in whether some individuals went up vs down in balance. For figure 2b, I suggest showing the full range of positive and negative changes, not the absolute value of the changes.

**Thank you for the suggestion. We initially opted for absolute aggression balance change as the model was more straightforward for the beta distribution. In the revised version, we changed the analysis and performed a robust mixed model on aggression balance. This is a cleaner statistical approach, but resulted in a similar outcome for the results, so our interpretation remained the same. Methods: P34. Results: P9.**

Figure 2b for Group Members contrasts changes in absolute aggression balance for the non-focal individuals in trials where top-ranked individuals were removed/reintroduced vs trials where middle/low ranked individuals were removed. It is worth noting that while the differences could be due to the rank of the individuals that were removed/reintroduced, they could also be due to the differences in the ranks of the group members themselves. When top-ranked individuals were reintroduced, the ‘group members’ were all middle/low ranked individuals, whereas when middle/low rank individuals were reintroduced, the ‘group members’ included the top ranked individuals.

**Thank you for raising this point. In earlier work, we found that group members reshuffled and updated their rank after perturbations. Rank is not a stable individual characteristic, it is constantly updated when new information (i.e., observing interactions within the group) becomes available.**

**We now explicitly acknowledge this limitation in the manuscript and discuss how capture-related effects could contribute to the observed differences between treatments (P16).**

Figure 2 contrasts aggression patterns before removal vs after reintroduction. I thought that the most interesting results would contrast how aggression patterns (for the non-focal birds) changed from before removal to after removal of focals, and then step 2 – from the removal phase to after reintroduction. Why are the patterns during the removal phase not shown? I strongly suggest that these be included, or even featured in the analyses.

**We thank the reviewer for the suggestion. As we included the focal birds, we only analyzed the periods when they were present in the group. In the revised version, we now include analysis for only the remaining group members (see new Fig2d). Methods: P35, results: P11.**

The analyses of shifts in aggression patterns for removals vs reintroductions of top-ranked vs middle/low ranked individuals were fascinating. Very interesting, and very cool. Figures 4 and 5 make it clear that both removals and reintroductions cause non-random shifts in aggression patterns. The results themselves were interesting, though not deeply surprising, but this is, to my knowledge, an innovative set of methods for quantifying these outcomes. I am, however, not convinced that the authors have done analyses that support their conclusion that ‘the rank of the perturbed individual affected the propensity and types of group-level aggression changes’ (from the Abstract). Figure 4 shows that transition probabilities (across the 3 types of patterns of aggression) LOOK different for perturbations of top-ranked vs middle-low ranked focal individuals, but I did not see where the authors actually did a statistical test of whether these transition probabilities differ statistically. They verbally summarized how shifts in aggression patterns differed from random for perturbations involving top-ranked focals and for those involving middle/low ranked individuals (visualized in figure 4), but unlike for figure 5, they did not show us the actual stats, and again, as far as I could tell, did not show us stats comparing the transition patterns for the perturbations of top-ranked vs middle/low ranked individuals. This looks like the classic issue of where a difference between two group in whether they exhibit statistically significant responses does not show whether they statistically significantly differed in their responses.

**Thank you for this suggestion. We had conducted this analysis but had not included it in the initial version of the manuscript. In response to your comment, we have now incorporated it. Specifically, we included a similar reference model as for the comparison between removals and reintroductions to test whether patterns differed significantly between top-ranked and low-ranked trials. We updated the methods (see P35-36), results (see P11) and figure (see Fig. 4) accordingly.**

While I liked the analyses of transitions among the three types of aggression patterns, it is worth noting that while breaking complex, continuous metrics (the pattern of aggression) into three types of patterns helps to tell a simple story, it might make the story simpler than reality. The definition of the 3 types of aggression patterns revolves around a metric for relative tendency to aggress individuals just below (close competitor), much farther below (bullying) vs relatively random with respect to relative rank. Each data set on patterns of aggression in a 3 day period could yield one or two metrics that summarize those relative tendencies and how consistent they are. Analyses could then be done on those metrics as opposed to on the simplified idea of three types of aggression patterns and transitions among them. A shift from one type to another – e.g., from CC to B could involve a small shift across the threshold that categorizes a pattern as CC to B, or a large change – but in the simplified categorization, these two changes, that might be quite different, would be both treated equally as a shift from CC to B. Would any results be qualitatively be altered by a more quantitative analysis?
**Thank you for raising this important point. The method to categorize the dominance patterns follows a structured procedure. The reference pattern (DH) is defined by a polygon based on the extremes of the 95% confidence intervals generated by a tuning parameter (i.e., the fraction of aggressive interactions that are randomly allocated).**

**Observed data are categorized relative to this polygon. When observed values fall within the polygon, the pattern is categorized as DH. Values below the polygon (i.e., lower position values than expected under the DH model) are categorized as close competitors, whereas values above the polygon (higher position than expected) are categorized as bullying.** **When focus values fall below those expected under random interactions, patterns are considered undefined. When patterns shift from DH to B or CC the values go in different directions of the polygons. The biggest shift is seen if patterns shift from CC to B or B to CC as the values have to move from one side of the polygon to the other.**

**Importantly, the polygon is not fixed in shape and size but changes according to observed values of each social group. This could result in different patterns with similar focus and position values across different groups. Thus, similar observed values can be categorized differently depending on group-specific aggression structure.**

**To account for group-specific aggression structure, we categorized the dominance patterns. In this revised version, we now included a visual example (period 1 group 2021) of how dominance patterns are categorized based on the reference model and the polygon around the 95% confidence intervals of the tuning parameter (SI6a) and the observed focus and position values in the supplemental material (SI6b, see also figure below). We furthermore clarified how dominance patterns are categorized in our methods section (P32-33).**

Figure SI6. Focus and position to categorize dominance patterns. Panel a) shows a visual example (period 1 group 2021) of how dominance patterns are categorized based on the reference model and the polygon around the 95% confidence intervals of the tuning parameter. Panel b) visualizes focus and position values for all 3-day periods and all social groups. This figure highlights that distinct social groups can have similar focus and position values which lead to different social dominance patterns due to underlying aggression structure.

These birds exhibit high fission-fusion dynamics in the wild. Does this mean that stable groups are rare – and does that mean that documenting social dynamics including dominance hierarchies in stable groups is ‘unnatural’?

**Wild monk parakeets show fission–fusion at the foraging/flock level, but they often maintain year-round colonial nesting groups with relatively stable core social units (pair). Fission–fusion may confer some resilience to minor membership changes, but removing or reintroducing a single individual is a qualitatively different type of perturbation. Our results suggest that even in a species accustomed to membership changes, perturbations of key individuals can still reorganize the social structure. The degree of resilience likely depends on which individuals are removed, group size, social context, and environmental conditions (e.g., resource availability). Thus far, there are no data on dominance hierarchies in the wild. We specified the limitation in our manuscript. P16**

Line 359 – for the definition of focus, the authors say that “a value closer to 1 means that the receivers of aggression are all two ranks down the hierarchy from the aggressors”. Why TWO ranks down, as opposed to ONE rank below?
**Thank you for noticing this. Focus with a value of 1 is that aggressors direct their aggression to receivers at similar rank positions below in the hierarchy. While a value of 0 means that aggression is more equally distributed across the hierarchy. We updated this now in the manuscript. P32**

Line 380 – the authors controlled for group size in their measure of aggression rate. Please explain how you did that? You could just divide by number of individuals, but there are various reasons why encounter rates might go up either faster or slower than linearly with increasing group size, depending on aggregation tendencies, masking etc.

**We controlled for group size by dividing by the number of individuals in the original submission. In the revised manuscript, we controlled for group size by including group size as a covariate and hours observed as an offset in the model to account for differences in encounter opportunities among groups. We furthermore updated this analysis to model raw interaction events instead of aggression rate and use the 3-day periods as fixed factor to assess stability over time with the pre-perturbation period as reference level. P33, P7.**
