# Supplementary material for: Social manipulations trigger shifts in group-level dominance patterns
Source: PLoS Comput Biol. 2026 Nov 5;22(11):e1014875. doi: 10.1371/journal.pcbi.1014875 (PMC13739146; doi:10.1371/journal.pcbi.1014875)
Supplement: Supplementary file 3 [file pcbi.1014875.s004.docx]

PCOMPBIOL-D-26-00608R1
Social manipulations trigger shifts in group-level dominance patterns
PLOS Computational Biology

Dear Dr. van der Marel,

Thank you for submitting your manuscript to PLOS Computational Biology. After careful consideration, we feel that it has merit but does not fully meet PLOS Computational Biology's publication criteria as it currently stands. Therefore, we invite you to submit a revised version of the manuscript that addresses the points raised during the review process.

Please submit your revised manuscript by Sep 08 2026 11:59PM. If you will need more time than this to complete your revisions, please reply to this message or contact the journal office at ploscompbiol@plos.org. When you're ready to submit your revision, log on to https://www.editorialmanager.com/pcompbiol/ and select the 'Submissions Needing Revision' folder to locate your manuscript file.

Please include the following items when submitting your revised manuscript:

* A letter that responds to each point raised by the editor and reviewer(s). You should upload this letter as a separate file labeled 'Response to Reviewers'. This file does not need to include responses to formatting updates and technical items listed in the 'Journal Requirements' section below.
* A marked-up copy of your manuscript that highlights changes made to the original version. You should upload this as a separate file labeled 'Revised Manuscript with Track Changes'.
* An unmarked version of your revised paper without tracked changes. You should upload this as a separate file labeled 'Manuscript'.

If you would like to make changes to your financial disclosure, competing interests statement, or data availability statement, please make these updates within the submission form at the time of resubmission. Guidelines for resubmitting your figure files are available below the reviewer comments at the end of this letter.

We look forward to receiving your revised manuscript.

Kind regards,

Minyu Feng, Ph.D.
Academic Editor
PLOS Computational Biology

Natalia Komarova
Section Editor
PLOS Computational Biology

# Reviewer 1

Reviewer #1: The authors have addressed the previous comments and the manuscript is overall improved. As I wrote in the first round of comments, I think this is a insightful study. However, I am still skeptical about a couple of things – these are mainly conceptual, and I am not sure that they are easy to address at this stage, but this does not mean that I am against publication of this manuscript! Specifically, I still think that “dominance pattern” is confusing. “Aggression patterns/dynamics/heuristics” would better reflect the trait of interest. In theory, there can be a downward heuristic or bullying or close competitor case in a group with more or less steep/linear/stable dominance hierarchy. I don’t want to be annoying, but I do not see the reason to have an extra term here which is also confusing. I understand that this build up the previous work from the group.

**We thank the reviewer for raising this point. We recognize that “aggression pattern/dynamics/heuristics” might on the surface appear less confusing, but we strongly feel that the particular pattern we are describing does require a specific term, and that there is a strong benefit of remaining consistent with the past literature to reduce confusion in continuity of this follow-up research. In the revised version, we further clarify exactly what we are referring to as a “dominance pattern” and stress the use of this as a specific defined term. Specifically, we have clarified this distinction in the revised manuscript by explicitly stating that dominance patterns concern the group-level rank-dependent directionality of aggression and are conceptually distinct from hierarchy linearity, steepness, and stability. (P3)**

**We also further clarify our use of other related terms, especially “aggression dynamics” to reduce chances of confusion with similar-sounding terms (i.e., in Table 1).**

In addition to that, I am not sure how useful is here the whole concept of ‘dominance pattern’. In my understanding, a minimal change in the direction of aggression can lead to a different pattern. So, is this biologically relevant? Wouldn’t be more relevant and straightforward to look at the dynamic changes of aggression towards more or less dominant groupmates – I mean, to avoid using the three patterns which make the analysis essentially categorical?

**We understand the reviewer’s standpoint to look at the dynamic changes of aggression towards more- and less-dominant group members. However, using focus and position (the 2 continuous parameters used to assess dominance patterns) directly would lead to different results. This occurs because the convex hull of the 95% CI of the reference model is defined separately for each group, meaning that similar observed focus and position values can be classified differently depending on group-specific structure. Besides mentioning this in the methods section ‘dominance pattern assessment’ (P34), we now also mention this point in the introduction. This part now reads as: “Dominance patterns are identified by comparing two parameters describing the distribution of aggression—its concentration on receivers at similar versus different rank distances and its position within the hierarchy—with values simulated from reference models (7). Because classification is based on each group’s observed values relative to its own reference distribution, similar parameter values may receive different classifications among groups.” (P4)**

**We also feel that a continuous approach would address a different question. Our objective was not to estimate how aggression changes continuously with relative dominance. The categorical approach allowed us to test whether groups differ in the type of rank-dependent aggression they exhibit, rather than only whether aggression increases or decreases with the rank difference between opponents.**

**We therefore consider the categorical analysis appropriate for the stated aims of the study and have retained it.**

As said, I don’t want to prevent publication of this ms due to these concerns.

Some minor comments:

In L94, ‘if aggression dynamics and dominance patterns are plastic’: what is the difference of these two?

**We appreciate this request for clarification. We have revised the sentence to distinguish aggression dynamics (the pattern of aggressive interactions among individuals, i.e., who aggresses whom, how often, and how aggression is distributed) from dominance patterns (the emergent, group-level structure that arises from those aggression dynamics). The text now reads as: “First, we hypothesized that if aggression dynamics and dominance patterns are plastic, our perturbations would result in individuals redirecting their aggression toward different subjects and to alter the rate and distribution of their aggressive interactions. These individual-level changes in who aggresses whom, and how often, would scale up to group-level responses, leading to shifts in the overall dominance pattern of the group compared to the pre-perturbation period.” (P5)**

I think that the last paragraph of the introduction can be improved:
L95 what means changing their aggression?

**We understand that this was not clear phrasing. This means altering the rate and distribution of their aggression. We now mention this instead of changing their aggression. P5**

L101 maybe rephrase to “...would have a greater impact because we considered these to be ‘key’ individuals with significant impact on social dynamics” ?

**We changed this sentence accordingly. P5**

L103 “ We predicted that removals would have a greater impact” than re-introductions?

**Yes, indeed. We changed this sentence accordingly. P5**

L119 aggression given/[aggression given + receive]

**We updated this but following the recommendation of reviewer 2 moved the definition to the first time mentioning it in the introduction and the glossary table. P5**

L139: why do focal individuals had aggression balance value closer to 1? For some individuals to have aggression balance value closer to 1, others need to have aggression balance value closer to 0. Did you remove only those with aggression balance value closer to 1 for a reason?

**We think we may have unclearly phrased this. We did not select individuals to remove or reintroduce (focal birds) with an aggression balance closer to 1 but rather individuals based on their dominance rank within the group. We specifically targeted top-ranked and lower-ranked individuals to test whether the rank of the perturbed individual influenced group-level responses. In monk parakeets, as in many species with dominance hierarchies, higher-ranked individuals naturally exhibit higher aggression balance (i.e., they aggress more than they receive), while lower-ranked individuals exhibit lower aggression balance. Therefore, the observation that focal birds had aggression balance values closer to 1 before removal reflects their rank position, not a selection bias. We changed the sentence to: “Focal birds (i.e., single individuals that were removed and reintroduced based on their rank, and not their aggression balance) went from an aggression balance value closer to 1 (only aggress) before removal to a value closer to 0 (only receive aggression) upon reintroduction (SI3, SI4).” P8**

L175: This applies to several places of the text, maybe you could use “Reintroduced birds” instead of “Focal birds” to make the text clearer? Because, here, for example, you refer to the agression received after re-introduction specifically.

**Thank you for the suggestion. We changed focal bird to reintroduced bird in this paragraph as this was the only paragraph we only analysed the moment of reintroduction instead of both removal and reintroduction.** **P10**

L186: these “6 shifts” are the 6 possible shift given the 3 difference “dominance patterns”?

**We meant the number of perturbation types. In the sentence above, we now included the total number of possible shifts (n = 6 total possible shifts). P11**

L322: In his section you could maybe mention what removal and re-introduction can represent in the wild. For example migration, predation etc.

**Thank you for the suggestion. We added a short paragraph in our ‘relating our results to the wild’ discussing that the removal of individuals could reflect events, such as death, predation, or dispersal of a group member whereas reintroductions could reflect the return of temporary absent group members. P16**

Figure 1: maybe explain the x-axis legend by specifying that you had 26 periods or sht.

**We now included in the figure caption that the x-axis represents the 3-day assessment periods to quantify aggression.** P21

References: I am not sure that they are updated in both the clean version and the version with the track-changes.

**Thank you for having checked the references. In the revised version, we made sure that the references were updated in both versions.**

# Reviewer 2

Reviewer #2: I have now re-reviewed the manuscript titled “Social manipulations trigger shifts in group-level dominance patterns”. I have greatly appreciated the effort in addressing my previous comments and the care in responding to them. At this stage, I only have a few minor comments that I’m sure the authors can easily address.

Samuele Ramellini
(I sign all of my reviews)
**Thank you Samuele. It was great to meet you at ISBE.**

L79: I quite liked the new structure of the initial paragraphs of the introduction. My only suggestion here is to make the start of this new paragraph a bit less abrupt. It comes a bit unexpected as the changes in social dynamics are much earlier. Maybe just a sentence here trying to link the concepts of changes in social structure and dominance hierarchy dynamics would help.

**Thank you. We now included an introductory sentence to this paragraph by tying the 2 together. This sentence now reads as: “One condition that may induce social plasticity in aggression and dominance patterns is changes in group membership, although such dynamics are relatively hard to follow under wild conditions.”.P4**

L93: again this introduction of group-level responses is a bit abrupt, as the concepts related to this are explained only at the very start of the intro. Maybe just a couple more words, expanding of what these group-level responses to the perturbations are.

**We included a sentence to explain the group-level responses. It now reads as: “First, we hypothesized that if aggression dynamics and dominance patterns are plastic, our perturbations would result in individuals redirecting their aggression toward different subjects and to alter the rate and distribution of their aggressive interactions. These individual-level changes in aggression would lead to shifts in the overall dominance pattern of the group compared to the pre-perturbation period.” P5**

L99: not super clear what the difference between a top-ranked trial and removal is.

**We understand where the confusion came from. We used trial and perturbation interchangeably. In the revised version, we changed ‘trials’ to ‘perturbations’ as we meant both removals and reintroductions with trial.**

L105: this is a major assumption of the manuscript, that individuals would have re-adjusted their ranks when the bird returns. Maybe provide some more context/citations on this?

**Thank you for the suggestion. We added the citation in the revised version. P5**

L108: I would move this sentence earlier in the paragraph, before actually using the two terms.

**We decided to remove this sentence and define what is a perturbation the first time mentioning it in our research question paragraph. The sentence now reads as: “Within four captive groups of monk parakeets (*Myiopsitta monachus*), we tested whether removals and reintroductions of focal individuals, i.e., a *perturbation* (Table 1), differing in *dominance rank* would 1) alter the aggression dynamics and 2) trigger dominance pattern shifts.” P4**

Table 1: not entirely clear what the social context is in the “social environment”. Also, some other terms are then used to describe glossary items (e.g. social structure used in dominance hierarchy) but are not defined. Maybe it’d be best to avoid these redundancies in the table and focus specifically on the terms that are key to the paper (e.g. dominance ones).
I would also consider carefully which terms to include in the glossary. Some of these are very general terms (social system) and are not really key to understand the rest of the paper. I’ll leave it to the authors to decide if and which ones to remove.

**Thank you for the suggestions. We removed the redundancies and only included terms focused on aggression, dominance, and the experiment. P6**

L123: probably worth giving a couple more details on the general framework of this model, how did this work? Just a couple sentences explaining the basics of the modelling procedure (I know that it’s in the methods, but being that they’re separated it’s probably worth reminding the reader).

**We agree that it is helpful to remind readers of the basic modelling framework in the introduction. We have added a short description explaining that the rank-based reference model classifies dominance patterns using two parameters—focus (the concentration of aggression across rank distances) and position (where aggression is focused relative to the aggressor’s rank)—and compares observed values to a downward-heuristic reference model with a tuning parameter that varies the proportion of random aggression. P4**

# Reviewer 3

Reviewer #3: I was reviewer 3 of the original manuscript. The authors have made important, useful changes to their manuscript, but I am not convinced that they addressed quite a few of my concerns. These were mostly somewhat technical points about analyses, but some important concerns could still benefit from more direct, explicit discussion in the paper.

**Thanks for your continued feedback, we are glad the changes in the last version made improvements and we worked to address your remaining concerns (below).**

My strongest concern revolved around the fact that the perturbations involved not just removal and subsequent reintroduction of focal birds (of different rank), but also, for the removal treatment, the capture and re-release of all birds. My comment was that it seems very conceivable that the capture, holding and then re-release of animals is disruptive enough to cause changes in aggression patterns. The authors note that they did not re-assess aggression patterns on the day of the capture and release, but it remains plausible that the effects of the capture-release disruption on aggression patterns carried over not just for the rest of that day, but into the near future, perhaps for the next week or two. The only clean way to control for that would be to capture and re-release all of the birds. The authors have added a paragraph that notes that because capture-release is likely disruptive, the fact that they did that for removals but not for reintroductions could explain the differences in effects of these two types of perturbations. I agree. However, my broader point is that the capture-release disruption per se could explain why the perturbations were associated with some changes in aggression patterns, regardless of whether a focal individual was removed or not. And, that it is conceivable that the differences between effects of removals of top-ranked versus lower-ranked individuals on the rest of the group is more about the nature of the rest of the group being systematically different when you only target the top-ranked individual versus when you remove a lower-ranked individual and leave the top ranked one. As noted in my earlier review, these concerns could be addressed by having controls where all birds were removed and re-released, which the authors did not do. The authors addressed this point in their reply to reviewers. They agreed that capture-release is very stressful, noting that this is why they did not run controls – but the stress of capture-release is my point exactly. They also noted that their aviary groups experienced other disturbances that did not alter dominance patterns. Of course, these other disturbances were NOT identical to the experimental disturbance, so to a critical reader, these disturbances are not a very good control. And, the authors noted that there were logistical constraints. They suggested that they acknowledge the limitation in the revised paper, but as far as I can tell, they only addressed the possibility that the stress of capture-release could explain, in part, differences between effects of removals vs reintroductions. They have not addressed the larger point that the capture-release disruption could explain the apparent effect of the removals! I suggest that the authors own up to this issue and address it explicitly and directly.
**We agree with the reviewer that capture, handling, and re-release are potentially disruptive and could affect aggression patterns beyond the removal day. This is indeed a limitation of our design for comparing how removals and reintroductions differentially affected the dominance patterns and aggression dynamics in these groups. We now explicitly acknowledge this limitation in the discussion. (P18)**

**The revised paragraph reads as follows: “It is important to note that responses to 1) removals and reintroductions, and 2) top-ranked versus lower-ranked perturbations should be compared with caveats. First, on removal days, all birds were trapped and handled, but on reintroduction days, only the reintroduced bird was handled to minimize stress (45–47) by reducing the number of trapping events for the group. Because of differences in methods, we could not fully separate treatment effects from possible effects of the trapping procedure itself, and it remains possible that handling stress contributed to some of the observed changes in aggression. However, since we analyzed aggression excluding the trapping day, the birds should have had sufficient time to recover from handling stress. Capture and handling typically elicit a strong but short-lived corticosterone response, with levels returning toward baseline within an hour and no consistent evidence of long-term behavioural disruption when animals are released promptly (46–48). The patterns we observed can therefore be reasonably, but not certainly, attributed to differential responses to the perturbations rather than just differences in handling. Nevertheless, we acknowledge that our study design does not provide a clean control for the capture–release procedure itself, and that this remains an important alternative explanation. To clearly differentiate between these effects would require two additional experiments in which (i) the entire group is captured and released on both removal and reintroduction days, and (ii) capture and release of the entire group is performed without experimental perturbations.”**\

My other moderately substantial point that was also raised by another reviewer involved the fact that these birds show fission-fusion dynamics where changes in group membership are likely larger and more frequent than what is experimentally addressed here: groups that are stable for quite some time, then 1 individual is removed, then 2-3 days with no further change, until that individual is reintroduced. It seems likely that this pattern does not resemble what happens in nature. I recognize that unnatural patterns of experimental manipulations in lab arenas are quite common – and that these experiments have yielded powerful insights on patterns of behavioral plasticity, so I don’t mean to harshly criticize this paper for that issue – but it could be worth discussing more explicitly. In their Reply, the authors say that they ‘specified this limitation…p16’ but there, they simply note the issue and suggest that it would be useful to do field work on this and other related species, but they could more explicitly note how their results might relate to what one might predict in more dynamic fission-fusion systems.

**We thank the reviewer for raising this concern. We now more explicitly discussed how our results might relate to species in the wild with fission-fusion dynamics. Our results from the captive groups are possibly a more conservative scenario of social plasticity, while wild groups may experience more pronounced changes in aggression dynamics and more shifts in dominance patterns. As groups in the wild may experience more frequent absences, variable durations in absences, and returns to different group configurations. This variability therefore may make wild groups potentially more flexible in their responses.**

**We have created a new section in the discussion ‘Relating our results to the wild’ where we discuss that captive responses may not directly predict wild responses; fission–fusion dynamics in the wild may differ from our experimental manipulation design in terms of number of individuals involved and duration of separation; and our results generate predictions about how wild groups might respond. P16, 17**

I also earlier noted that when they contrast the effects of removing a top-ranked vs lower-ranked individual on the rest of the group, any differences could be due to differences in the type of individual removed, but the treatments also differ in the type of individuals that were NOT removed. This is a perhaps subtle, but simple enough point to discuss, but the authors Reply did not, in my view, really address it, and did not apparently address it in the revised paper.

**Our apologies for the fact that we did not properly address your point in the former version. We have now acknowledged this caveat in our limitations section, where we discuss that differences in group responses between these treatments could reflect either the disproportionate influence of losing a ‘key’ individual or the different social contexts experienced by the remaining members. But that our study design does not allow these 2 effects to be separated. And we explicitly stated what type of experiments are necessary to disentangle these effects. P19**

I am satisfied and pleased that the authors changed their analysis and presentation of aggression balance from absolute values of changes to a metric that separates between increases vs decreases in aggression balance. And, I like their additions to figure 4, and I think I understand what the authors show in figure S16.

**Thank you.**

However, I could not tell whether and how their ‘robust mixed model’ accounts for ceiling-floor effects on the possible values of aggression balance. I suggest adding a few words on this in their Methods.

**We thank the reviewer for raising the issue of ceiling–floor effects on aggression balance. Aggression balance is bounded between 0 and 1, and the change in aggression balance between periods is therefore bounded between −1 and 1. Our robust linear mixed‑effects model treats the change in aggression balance as a continuous response and does not explicitly model these bounds, although robust estimation down‑weights outlying observations, including those near the boundaries. As we only had 3% of observations near the boundaries (threshold of -0.9 and 0.9), we believe that the robust model was appropriate. We now mention this in the methods section ‘aggression dynamics’ (P35).**

**We still conducted a check using a binomial generalized linear mixed model on the raw aggression counts (aggression given vs received). This model respects the 0–1 bounds of aggression balance. The results regarding the effects of focal rank, focal status, and their interaction were qualitatively similar to those from the robust linear mixed model: focal birds showed a stronger reduction in aggression after reintroduction than nonfocal birds, and this difference was more pronounced in top‑ranked than in lower‑ranked perturbations. We have added a brief description of this extra analysis (P35) and its results (P10), and included the results of the model in Supplemental Material SI4.1.**

Small point. Line 99 – predicts greater aggression balance differences, but I don’t think the term ‘aggression balance’ has been defined yet.

**That was true. In this revised version, we included the term in the glossary, defined it in this sentence (P6), and removed it when we defined it in the result section (P7).**

I did not find the time to comment on details and wording that could improve clarity, but am pleased to see that other reviewers provided numerous suggestions.
