# Supplementary material for: Social manipulations trigger shifts in group-level dominance patterns
Source: PLoS Comput Biol. 2026 Nov 5;22(11):e1014875. doi: 10.1371/journal.pcbi.1014875 (PMC13739146; doi:10.1371/journal.pcbi.1014875)
Supplement: Peer review history

# Peer review: Decision Letter 0

**DOI:** 10.1371/journal.pcbi.1014875.r001

**Authors:** Natalia Komarova, Minyu Feng

9 Apr 2026

Social manipulations trigger shifts in group-level dominance patterns

PLOS Computational Biology

Dear Dr. van der Marel,

Thank you for submitting your manuscript to PLOS Computational Biology. After careful consideration, we feel that it has merit but does not fully meet PLOS Computational Biology's publication criteria as it currently stands. Therefore, we invite you to submit a revised version of the manuscript that addresses the points raised during the review process.

Please submit your revised manuscript by Jun 09 2026 11:59PM. If you will need more time than this to complete your revisions, please reply to this message or contact the journal office at ploscompbiol@plos.org. When you're ready to submit your revision, log on to https://www.editorialmanager.com/pcompbiol/ and select the 'Submissions Needing Revision' folder to locate your manuscript file.

Please include the following items when submitting your revised manuscript:

* A letter that responds to each point raised by the editor and reviewer(s). You should upload this letter as a separate file labeled 'Response to Reviewers'. This file does not need to include responses to formatting updates and technical items listed in the 'Journal Requirements' section below.

* A marked-up copy of your manuscript that highlights changes made to the original version. You should upload this as a separate file labeled 'Revised Manuscript with Track Changes'.

* An unmarked version of your revised paper without tracked changes. You should upload this as a separate file labeled 'Manuscript'.

If you would like to make changes to your financial disclosure, competing interests statement, or data availability statement, please make these updates within the submission form at the time of resubmission. Guidelines for resubmitting your figure files are available below the reviewer comments at the end of this letter

We look forward to receiving your revised manuscript.

Kind regards,

Minyu Feng, Ph.D.

Academic Editor

PLOS Computational Biology

Natalia Komarova

Section Editor

PLOS Computational Biology

**Additional Editor Comments :**

Our experts are broadly positive about the question, system, and methods, but converge on the need for much clearer writing and conceptual consistency, and more rigorous and transparent treatment of design limitations and statistical comparisons, especially around the role of capture, handling, and rank effects on aggression patterns. Please address all the issues before resubmission.

**Journal Requirements:**

1) Please provide an Author Summary. This should appear in your manuscript between the Abstract (if applicable) and the Introduction, and should be 150-200 words long. The aim should be to make your findings accessible to a wide audience that includes both scientists and non-scientists. Sample summaries can be found on our website under Submission Guidelines:

https://journals.plos.org/ploscompbiol/s/submission-guidelines#loc-parts-of-a-submission

2) We do not publish any copyright or trademark symbols that usually accompany proprietary names, eg ©,  ®, or TM  (e.g. next to drug or reagent names). Therefore please remove all instances of trademark/copyright symbols throughout the text, including:

- ® on page: 20.

3) Please upload all main figures as separate Figure files in .tif or .eps format. For more information about how to convert and format your figure files please see our guidelines:

https://journals.plos.org/ploscompbiol/s/figures

4) We have noticed that you have uploaded Supporting Information files, but you have not included a list of legends. Please add a full list of legends for your Supporting Information files after the references list.

5) Please ensure that the funders and grant numbers match between the Financial Disclosure field and the Funding Information tab in your submission form. Note that the funders must be provided in the same order in both places as well.

Note: If the reviewer comments include a recommendation to cite specific previously published works, please review and evaluate these publications to determine whether they are relevant and should be cited. There is no requirement to cite these works unless the editor has indicated otherwise.

**Reviewers' comments:**

Reviewer's Responses to Questions

Reviewer #1: I think this is an interesting manuscript presenting some clearly designed experiments, and providing useful insights for the study of dominance and aggression. Yet, I think that the readability can be significantly improved, and certain points are important to be clarified.

First, I think that the authors can improve the consistency of the terminology which currently makes the text hard to follow (e.g., aggression dynamics vs aggression decisions vs dominance dynamics OR aggression vs agonistic interaction OR dominance rank vs social role vs power score).

Second, this article is heavily based on a previous one on dominance patters and aggression heuristics (Hobson et al 2021). I think that the authors need to more thoroughly explain the differences of the dominance patterns in this new article, so the reader can smoothly follow the reasoning. While there are some details in the methods, I think that some of it can be introduced earlier.

Abstract: I know that we are often supposed to have a fancy 1-2 sentence intro in our abstracts highlighting an important gap of knowledge. I think that the first three sentences of the abstract do not flow well. Mentioning dynamic social systems, social plasticity, hierarchies, aggression etc makes them hard to follow. The authors may want to simplify them. Then, again, in L30 “To investigate factors affecting dominance patterns” starts quite general. The authors could simply write directly sth like “We studied the link…”. I do not see why to refer to “dominance patterns” when one can simply say sth like “rank dependent aggression”, or “aggression heuristics” that has been used in the past

Hobson et al 2021 Aggression heuristics underlie animal dominance hierarchies and provide evidence of group-level social information

Smit & Robbins 2025 Risk-taking incentives predict aggression heuristics in female gorillas

Finally, the last sentence of the abstract, I feel that is again introducing new concepts without fully explaining the relevance. At the end of the discussion it is clearer.

L47: why “especially in groups structured by aggression and dominance hierarchies “ ?

L49: I think that again this “especially… rather…” needs explanation.

L51: I find “consensus among group members on who to aggress” unclear. I assume the authors mean depending on rank? I think that using a term like “rank-based aggression” or sth similar would be more clear.

L52: I understand “dominance” as something that characterizes relationships, and aggression something that can be instantaneous, characterizing interactions. Thus, I would not use aggression and dominance patterns as equivalent.

L65: Why using “social role (dominance rank)” and not directly “dominance rank”

L67: “are highly social parrots” ? (plural)

L70: Here you first mention “aggression dynamics” which, I think, is more accurate.

L79: So, reintroduction of a previously high ranking individual, would take things back in the previous hierarchy smoothly or the reintroduced individual would occupy a rank at the bottom of the hierarhcy?

L85: I guess this is an expected comment: why 3 days? I understand that the authors need a threshold, but maybe a little explanation would help.

L87: Does “power score” mean simplify “dominance rank”? If yes, then maybe simply use this term? If not, using the term “power” here for first time, without defining it, is confusing, as it is a ‘parent category’ of dominance (for example: Davidian et al 2022 The eco-evolutionary landscape of power relationships between males and females)

L91: Here is another new term “agonistic”. Is this the same with “aggressive”? If not explanation of the difference is missing; if yes, why use another term and not being consistent?

L111: Put all this numbers in a parenthesis at the end to improve readability? (0.26 ± 0.02... vs 0.09…)

L114: I am a bit confused here on why the authors compare top-ranked focal birds with group members from middle/low-ranked trials (and middle/low-ranked focal birds with group members from top-ranked trials), instead of comparing focal birds of different rank as well as remaining group memebers of different rank.

L117: “depending on their” instead of “compared to its”?

L123: In almost all text the authors use “middle/low”. I think this is a bit unclear if they refer to two different categories for which the results were always similar, or one category. If the latter, maybe use one word?

L53: Referring to dominance patterns here made me go back to the first mention of those. In L53 the authors say that “consensus among group members on who to aggress...a dominance pattern can emerge”. But later on, in L129, they report percentages which, I assume, mean that 45% of all aggression was towards “any individual lower-ranked than self”, 33% was “towards much lower-ranked individuals” etc. So, is the dominance pattern a group trait (consensus OR) or an individual trait? Finally, aren’t the close competitors and bullying patterns subcategories of downward heuristic?

L179-182: Some of 1,2,3 include methods and other results.

L187: If it is “the history of social interactions”, why the re-introduced individuals don’t reoccupy they previous positions smoothly? Seems like there is a temporal aspect. You somehow discuss that later on, but maybe the “history of insteraction” is not the actual determinant?

L196: I do not understand the “shifts within the same group from multiple groups”

L220: Some other relevant refs to the point that I found. I am not asking the authors to cite this work. I just found these articles quite relevant.

Franz et al 2015 Knockouts of high-ranking males have limited impact on baboon social networks

Ewbank & Meese 2010 Aggressive behaviour in groups of domesticated pigs on removal and return of individuals

Piefke et al 2021 Social network stability is impacted by removing a dominant male in replicate dominance hierarchies of a cichlid fish

L232: I am wondering if a shift in dominance pattern necessarily means “social destabilization”.

L380: If I understand correctly, aggression balance is an individual property, while you start the paragraph referring to “each group and pattern assessment period”

I admit that I didn’t manage to focus well on the methods, because of the inconsistencies in concepts etc I highlighted above. However, I think that the methods had generally a better flow.

Reviewer #2: This paper entitled “Social manipulations trigger shifts in group-level dominance patterns” addresses changes in dominance patterns following experimental removals or reintroductions of individuals of different ranks. I quite appreciate the depth of detail in the manuscript and there is a great wealth of analyses. I have highlighted below some of my concerns. I think the paper would greatly benefit from an overall increase of sharpness. I find it at times quite hard to read and follow, there are a lot of different terms and a bit of jargon that makes the reader continuously have to go back and forth for definitions. I think that addressing some of the concerns I raise in this review could really help. I hope that I can make it clear why I have come to this conclusion, and that the authors can address these in the revisions (or better explain their work if I have misunderstood).

Samuele Ramellini

(I sign all of my reviews)

Relevance: My main question is related to why this is relevant in nature. While I appreciate the mention of fission fusion dynamics, I was honestly expecting much more discussion about how these mechanisms have emerged and how the specific social system of the species might have favoured/fixed these mechanisms. The authors seem to suggest that group composition should be robust to changes. However, it seems that dominance is changing all the time, because of perturbations but also just randomly. How does this tie in with the social system of the species? How could this be extended to other species with the same social structure? The last few sentences seem to be pointing a little bit in this direction, but these are just left at the end of the manuscript, not really introduced or discussed anywhere else.

Focus of introduction: I found the intro is very based on individual level changes while the rest of the paper actually focuses strongly on group level dominance pattern changes. Maybe referring to “emergent” patterns and explore the literature on this a little bit more would ease the transition and help the reader framing the results.

Perturbation & other definitions: I find the combination of removals and reintroductions into “perturbations” very confusing. I find hard to understand what the predictions are referring to (e.g. L70-L71) and these could be made into sharper and directional predictions for either of the removals or reintroductions.

Rule-following is mentioned only in Fig 2, and never really defined in the text. These are also previously referred as downward heuristics (L53).

There are a lot of terms (e.g. focal individual, rule following, dominance patterns) that are briefly defined but used extensively. A lot of these terms can be redefined or restated as that would really help in following the language across the manuscript.

Interval experiments: The interval between perturbations seems to be really short. For example, there are only a few days between the reintroduction of an individual and the removal of another one (I’m referring to the interval between the 17-days trials). In Fig. 1c, it seems that after the first reintroduction of the top-ranked individuals, there is an increase in the aggression rate. But after one three-day assessment period, the middle ranked are excluded, so it’s not clear if the dynamics went back to being comparable to pre-perturbation levels.

Finally, how biologically relevant is this interval? Is there any evidence in nature that these events are this regular and this intense (one removal/reintroduction every two weeks)?

Stability of aggression rate: In L99, I agree that this rate is clearly higher, but it seems that this is the baseline level for that group (the pre-perturbation levels in the first two assessments are exactly on the average). It seems that these two groups had just more aggressive background levels. How does this affect the results? How are the four groups compared in this sense?

Minor comments

L34. I would consider reversing the order of 1 and 2. It seems like the more important result is the second.

L36-37. Worth specifying how these reintroductions affected group-level hierarchical organisation.

L65-67. It’s a bit confusing referring to “individuals” in these sentences. In the first “individual” of L65, I guess perturbation is referring to the removed individual (in which case I would just call it removal). While in the second case, the change in “individual aggression decision-making” is referring to the remaining individuals (the not removed). Maybe restructuring this sentence to reflect these specifics would help solving the confusion. Also, maybe worth going back to the “focal” language.

L73. I’m not quite sure what “possible drivers” mean here.

L74. What do the authors mean with more changes? More in terms of number, in terms of intensity of changes? Please clarify.

L79. This last prediction is a bit confusing. Is it predicting that the dominance pattern would return to the pre-removal level? L81, is the “their” referring to the reintroduced bird or the remaining individuals?

L86-86: Are there events that include more than one individual? Is there any reason to specify that the authors only use directional aggression in L86? Is there any non-directional aggression?

Also, in Fig 2a, why is there only one focal individual highlighted? Shouldn’t it be three?

L91. This seems a bit repetitive from the previous paragraph.

L94. I guess this refers to the removal of one top-ranked bird per group as compared to 3 top-ranked birds in 2022.

L101: I’m wondering whether instead of the absolute change in aggression balance, it’d be worth showing the change in general. Are these birds experiencing more or less aggression?

L219: this sentence seems to suggest group coordination in changes. I would refer to these as emergent patterns rather than group shifts. There is not much testing of group decision making in this manuscript.

Reviewer #3: I liked the system, the main questions and the experimental paradigm of social perturbations involving removal and reintroductions of high-ranked vs lower-ranked individuals. I particularly liked the analyses examining transition probabilities across three types of rank-based aggression patterns and how these transitions might differ for perturbations of top-ranked vs middle/low ranked individuals (versus control situations with no perturbations) and for removals vs reintroductions.

I worry that the experimental design had no controls for a removal, all birds were captured and then re-released except for the birds that were ‘removal’ birds. A control would be to capture them all, and then re-release them all. It seems very conceivable that the act of capturing and re-releasing birds causes changes in dominance and aggression patterns. Differences between effects of temporarily removing high ranked vs lower ranked birds could be due to differing effects of the capture/release perturbation per se on high ranked birds (in the treatment that removed lower ranked birds) vs capture/release per se on lower ranked birds (in the treatment that removed higher ranked birds). Differences between removals vs reintroductions could obviously be strongly influenced by the fact that the reintroductions do not involve capture and release of all other individuals. This strikes me as an important flaw in the experimental design that, at minimum, should be discussed head-on, or in a devil’s advocate view, significantly reduces confidence in results.

Figure 2b shows absolute aggression balance changes (aggression balance upon reintroduction minus aggression balance before removal) for focal birds vs group members, separately for top ranked vs lower ranked birds. While these figures look interesting, to understand what is happening, it would be more useful to see each individual’s balance before removal AND upon reintroduction, with lines joining these values. Ceiling/floor effects (balance scores go from 0 to 1) can obviously affect how much an individual’s balance score could potentially change in one direction or the other, and more importantly, patterns of change (which ones went up versus down) are obviously obscured when one shows the absolute value of changes. Are we really interested in the absolute value of the changes? I would be much more interested in whether some individuals went up vs down in balance. For figure 2b, I suggest showing the full range of positive and negative changes, not the absolute value of the changes.

Figure 2b for Group Members contrasts changes in absolute aggression balance for the non-focal individuals in trials where top-ranked individuals were removed/reintroduced vs trials where middle/low ranked individuals were removed. It is worth noting that while the differences could be due to the rank of the individuals that were removed/reintroduced, they could also be due to the differences in the ranks of the group members themselves. When top-ranked individuals were reintroduced, the ‘group members’ were all middle/low ranked individuals, whereas when middle/low rank individuals were reintroduced, the ‘group members’ included the top ranked individuals.

Figure 2 contrasts aggression patterns before removal vs after reintroduction. I thought that the most interesting results would contrast how aggression patterns (for the non-focal birds) changed from before removal to after removal of focals, and then step 2 – from the removal phase to after reintroduction. Why are the patterns during the removal phase not shown? I strongly suggest that these be included, or even featured in the analyses.

The analyses of shifts in aggression patterns for removals vs reintroductions of top-ranked vs middle/low ranked individuals were fascinating. Very interesting, and very cool. Figures 4 and 5 make it clear that both removals and reintroductions cause non-random shifts in aggression patterns. The results themselves were interesting, though not deeply surprising, but this is, to my knowledge, an innovative set of methods for quantifying these outcomes. I am, however, not convinced that the authors have done analyses that support their conclusion that ‘the rank of the perturbed individual affected the propensity and types of group-level aggression changes’ (from the Abstract). Figure 4 shows that transition probabilities (across the 3 types of patterns of aggression) LOOK different for perturbations of top-ranked vs middle-low ranked focal individuals, but I did not see where the authors actually did a statistical test of whether these transition probabilities differ statistically. They verbally summarized how shifts in aggression patterns differed from random for perturbations involving top-ranked focals and for those involving middle/low ranked individuals (visualized in figure 4), but unlike for figure 5, they did not show us the actual stats, and again, as far as I could tell, did not show us stats comparing the transition patterns for the perturbations of top-ranked vs middle/low ranked individuals. This looks like the classic issue of where a difference between two group in whether they exhibit statistically significant responses does not show whether they statistically significantly differed in their responses.

While I liked the analyses of transitions among the three types of aggression patterns, it is worth noting that while breaking complex, continuous metrics (the pattern of aggression) into three types of patterns helps to tell a simple story, it might make the story simpler than reality. The definition of the 3 types of aggression patterns revolves around a metric for relative tendency to aggress individuals just below (close competitor), much farther below (bullying) vs relatively random with respect to relative rank. Each data set on patterns of aggression in a 3 day period could yield one or two metrics that summarize those relative tendencies and how consistent they are. Analyses could then be done on those metrics as opposed to on the simplified idea of three types of aggression patterns and transitions among them. A shift from one type to another – e.g., from CC to B could involve a small shift across the threshold that categorizes a pattern as CC to B, or a large change – but in the simplified categorization, these two changes, that might be quite different, would be both treated equally as a shift from CC to B. Would any results be qualitatively be altered by a more quantitative analysis?

These birds exhibit high fission-fusion dynamics in the wild. Does this mean that stable groups are rare – and does that mean that documenting social dynamics including dominance hierarchies in stable groups is ‘unnatural’?

Line 359 – for the definition of focus, the authors say that “a value closer to 1 means that the receivers of aggression are all two ranks down the hierarchy from the aggressors”. Why TWO ranks down, as opposed to ONE rank below?

Line 380 – the authors controlled for group size in their measure of aggression rate. Please explain how you did that? You could just divide by number of individuals, but there are various reasons why encounter rates might go up either faster or slower than linearly with increasing group size, depending on aggregation tendencies, masking etc.

**********

**Have the authors made all data and (if applicable) computational code underlying the findings in their manuscript fully available?**

The PLOS Data policy requires authors to make all data and code underlying the findings described in their manuscript fully available without restriction, with rare exception (please refer to the Data Availability Statement in the manuscript PDF file). The data and code should be provided as part of the manuscript or its supporting information, or deposited to a public repository. For example, in addition to summary statistics, the data points behind means, medians and variance measures should be available. If there are restrictions on publicly sharing data or code —e.g. participant privacy or use of data from a third party—those must be specified.

Reviewer #1: None

Reviewer #2: Yes

Reviewer #3: Yes

**********

PLOS authors have the option to publish the peer review history of their article (what does this mean?). If published, this will include your full peer review and any attached files.

If you choose “no”, your identity will remain anonymous but your review may still be made public.

**Do you want your identity to be public for this peer review?**  For information about this choice, including consent withdrawal, please see our Privacy Policy.

Reviewer #1: No

Reviewer #2: No

Reviewer #3: No

[NOTE: If reviewer comments were submitted as an attachment file, they will be attached to this email and accessible via the submission site. Please log into your account, locate the manuscript record, and check for the action link "View Attachments". If this link does not appear, there are no attachment files.]

**Figure resubmission:**

After uploading your figures to PLOS’s NAAS tool - https://ngplosjournals.pagemajik.ai/artanalysis, NAAS will process the files provided and display the results in the "Uploaded Files" section of the page as the processing is complete. If the uploaded figures meet our requirements (or NAAS is able to fix the files to meet our requirements), the figure will be marked as "fixed" above. If NAAS is unable to fix the files, a red "failed" label will appear above. When NAAS has confirmed that the figure files meet our requirements, please download the file via the download option, and include these NAAS processed figure files when submitting your revised manuscript.

**Reproducibility:**

To enhance the reproducibility of your results, we recommend that authors of applicable studies deposit laboratory protocols in protocols.io, where a protocol can be assigned its own identifier (DOI) such that it can be cited independently in the future. Additionally, PLOS ONE offers an option to publish peer-reviewed clinical study protocols. Read more information on sharing protocols at https://plos.org/protocols?utm_medium=editorial-email&utm_source=authorletters&utm_campaign=protocols

# Author comment: Author response to Decision Letter 1

**DOI:** 10.1371/journal.pcbi.1014875.r002

11 Jun 2026

Attachment Submitted filename: PCOMPBIOL_response letter_v4.docx

# Peer review: Decision Letter 1

**DOI:** 10.1371/journal.pcbi.1014875.r003

**Authors:** Natalia Komarova, Minyu Feng

9 Jul 2026

Social manipulations trigger shifts in group-level dominance patterns

PLOS Computational Biology

Dear Dr. van der Marel,

Thank you for submitting your manuscript to PLOS Computational Biology. After careful consideration, we feel that it has merit but does not fully meet PLOS Computational Biology's publication criteria as it currently stands. Therefore, we invite you to submit a revised version of the manuscript that addresses the points raised during the review process.

Please submit your revised manuscript by Sep 08 2026 11:59PM. If you will need more time than this to complete your revisions, please reply to this message or contact the journal office at ploscompbiol@plos.org. When you're ready to submit your revision, log on to https://www.editorialmanager.com/pcompbiol/ and select the 'Submissions Needing Revision' folder to locate your manuscript file.

Please include the following items when submitting your revised manuscript:

* A letter that responds to each point raised by the editor and reviewer(s). You should upload this letter as a separate file labeled 'Response to Reviewers'. This file does not need to include responses to formatting updates and technical items listed in the 'Journal Requirements' section below.

* A marked-up copy of your manuscript that highlights changes made to the original version. You should upload this as a separate file labeled 'Revised Manuscript with Track Changes'.

* An unmarked version of your revised paper without tracked changes. You should upload this as a separate file labeled 'Manuscript'.

Kind regards,

Minyu Feng, Ph.D.

Academic Editor

PLOS Computational Biology

Natalia Komarova

Section Editor

PLOS Computational Biology

**Additional Editor Comments (if provided):**

**Reviewers' comments:**

Reviewer's Responses to Questions

**Comments to the Authors:**

**Please note here if the review is uploaded as an attachment.**

Reviewer #1: The authors have addressed the previous comments and the manuscript is overall improved. As I wrote in the first round of comments, I think this is a insightful study. However, I am still skeptical about a couple of things – these are mainly conceptual, and I am not sure that they are easy to address at this stage, but this does not mean that I am against publication of this manuscript! Specifically, I still think that “dominance pattern” is confusing. “Aggression patterns/dynamics/heuristics” would better reflect the trait of interest. In theory, there can be a downward heuristic or bullying or close competitor case in a group with more or less steep/linear/stable dominance hierarchy. I don’t want to be annoying, but I do not see the reason to have an extra term here which is also confusing. I understand that this build up the previous work from the group.

In addition to that, I am not sure how useful is here the whole concept of ‘dominance pattern’. In my understanding, a minimal change in the direction of aggression can lead to a different pattern. So, is this biologically relevant? Wouldn’t be more relevant and straightforward to look at the dynamic changes of aggression towards more or less dominant groupmates – I mean, to avoid using the three patterns which make the analysis essentially categorical?

As said, I don’t want to prevent publication of this ms due to these concerns.

Some minor comments:

In L94, ‘if aggression dynamics and dominance patterns are plastic’: what is the difference of these two?

I think that the last paragraph of the introduction can be improved:

L95 what means changing their aggression?

L101 maybe rephrase to “...would have a greater impact because we considered these to be ‘key’ individuals with significant impact on social dynamics” ?

L103 “ We predicted that removals would have a greater impact” than re-introductions?

L119 aggression given/[aggression given + receive]

L139: why do focal individuals had aggression balance value closer to 1? For some individuals to have aggression balance value closer to 1, others need to have aggression balance value closer to 0. Did you remove only those with aggression balance value closer to 1 for a reason?

L175: This applies to several places of the text, maybe you could use “Reintroduced birds” instead of “Focal birds” to make the text clearer? Because, here, for example, you refer to the agression received after re-introduction specifically.

L186: these “6 shifts” are the 6 possible shift given the 3 difference “dominance patterns”?

L322: In his section you could maybe mention what removal and re-introduction can represent in the wild. For example migration, predation etc.

Figure 1: maybe explain the x-axis legend by specifyin that you had 26 periods or sht.

References: I am not sure that they are updated in both the clean version and the version with the track-changes.

Reviewer #2: I have now re-reviewed the manuscript titled “Social manipulations trigger shifts in group-level dominance patterns”. I have greatly appreciated the effort in addressing my previous comments and the care in responding to them. At this stage, I only have a few minor comments that I’m sure the authors can easily address.

Samuele Ramellini

(I sign all of my reviews)

L79: I quite liked the new structure of the initial paragraphs of the introduction. My only suggestion here is to make the start of this new paragraph a bit less abrupt. It comes a bit unexpected as the changes in social dynamics are much earlier. Maybe just a sentence here trying to link the concepts of changes in social structure and dominance hierarchy dynamics would help.

L93: again this introduction of group-level responses is a bit abrupt, as the concepts related to this are explained only at the very start of the intro. Maybe just a couple more words, expanding of what these group-level responses to the perturbations are.

L99: not super clear what the difference between a top-ranked trial and removal is.

L105: this is a major assumption of the manuscript, that individuals would have re-adjusted their ranks when the bird returns. Maybe provide some more context/citations on this?

L108: I would move this sentence earlier in the paragraph, before actually using the two terms.

Table 1: not entirely clear what the social context is in the “social environment”. Also, some other terms are then used to describe glossary items (e.g. social structure used in dominance hierarchy) but are not defined. Maybe it’d be best to avoid these redundancies in the table and focus specifically on the terms that are key to the paper (e.g. dominance ones).

I would also consider carefully which terms to include in the glossary. Some of these are very general terms (social system) and are not really key to understand the rest of the paper. I’ll leave it to the authors to decide if and which ones to remove.

L123: probably worth giving a couple more details on the general framework of this model, how did this work? Just a couple sentences explaining the basics of the modelling procedure (I know that it’s in the methods, but being that they’re separated it’s probably worth reminding the reader).

Reviewer #3: I was reviewer 3 of the original manuscript. The authors have made important, useful changes to their manuscript, but I am not convinced that they addressed quite a few of my concerns. These were mostly somewhat technical points about analyses, but some important concerns could still benefit from more direct, explicit discussion in the paper.

My strongest concern revolved around the fact that the perturbations involved not just removal and subsequent reintroduction of focal birds (of different rank), but also, for the removal treatment, the capture and re-release of all birds. My comment was that it seems very conceivable that the capture, holding and then re-release of animals is disruptive enough to cause changes in aggression patterns. The authors note that they did not re-assess aggression patterns on the day of the capture and release, but it remains plausible that the effects of the capture-release disruption on aggression patterns carried over not just for the rest of that day, but into the near future, perhaps for the next week or two. The only clean way to control for that would be to capture and re-release all of the birds. The authors have added a paragraph that notes that because capture-release is likely disruptive, the fact that they did that for removals but not for reintroductions could explain the differences in effects of these two types of perturbations. I agree. However, my broader point is that the capture-release disruption per se could explain why the perturbations were associated with some changes in aggression patterns, regardless of whether a focal individual was removed or not. And, that it is conceivable that the differences between effects of removals of top-ranked versus lower-ranked individuals on the rest of the group is more about the nature of the rest of the group being systematically different when you only target the top-ranked individual versus when you remove a lower-ranked individual and leave the top ranked one. As noted in my earlier review, these concerns could be addressed by having controls where all birds were removed and re-released, which the authors did not do. The authors addressed this point in their reply to reviewers. They agreed that capture-release is very stressful, noting that this is why they did not run controls – but the stress of capture-release is my point exactly. They also noted that their aviary groups experienced other disturbances that did not alter dominance patterns. Of course, these other disturbances were NOT identical to the experimental disturbance, so to a critical reader, these disturbances are not a very good control. And, the authors noted that there were logistical constraints. They suggested that they acknowledge the limitation in the revised paper, but as far as I can tell, they only addressed the possibility that the stress of capture-release could explain, in part, differences between effects of removals vs reintroductions. They have not addressed the larger point that the capture-release disruption could explain the apparent effect of the removals! I suggest that the authors own up to this issue and address it explicitly and directly.

My other moderately substantial point that was also raised by another reviewer involved the fact that these birds show fission-fusion dynamics where changes in group membership are likely larger and more frequent than what is experimentally addressed here: groups that are stable for quite some time, then 1 individual is removed, then 2-3 days with no further change, until that individual is reintroduced. It seems likely that this pattern does not resemble what happens in nature. I recognize that unnatural patterns of experimental manipulations in lab arenas are quite common – and that these experiments have yielded powerful insights on patterns of behavioral plasticity, so I don’t mean to harshly criticize this paper for that issue – but it could be worth discussing more explicitly. In their Reply, the authors say that they ‘specified this limitation…p16’ but there, they simply note the issue and suggest that it would be useful to do field work on this and other related species, but they could more explicitly note how their results might relate to what one might predict in more dynamic fission-fusion systems.

I also earlier noted that when they contrast the effects of removing a top-ranked vs lower-ranked individual on the rest of the group, any differences could be due to differences in the type of individual removed, but the treatments also differ in the type of individuals that were NOT removed. This is a perhaps subtle, but simple enough point to discuss, but the authors Reply did not, in my view, really address it, and did not apparently address it in the revised paper.

I am satisfied and pleased that the authors changed their analysis and presentation of aggression balance from absolute values of changes to a metric that separates between increases vs decreases in aggression balance. And, I like their additions to figure 4, and I think I understand what the authors show in figure S16.

However, I could not tell whether and how their ‘robust mixed model’ accounts for ceiling-floor effects on the possible values of aggression balance. I suggest adding a few words on this in their Methods.

Small point. Line 99 – predicts greater aggression balance differences, but I don’t think the term ‘aggression balance’ has been defined yet.

I did not find the time to comment on details and wording that could improve clarity, but am pleased to see that other reviewers provided numerous suggestions.

**********

**Have the authors made all data and (if applicable) computational code underlying the findings in their manuscript fully available?**

The PLOS Data policy requires authors to make all data and code underlying the findings described in their manuscript fully available without restriction, with rare exception (please refer to the Data Availability Statement in the manuscript PDF file). The data and code should be provided as part of the manuscript or its supporting information, or deposited to a public repository. For example, in addition to summary statistics, the data points behind means, medians and variance measures should be available. If there are restrictions on publicly sharing data or code —e.g. participant privacy or use of data from a third party—those must be specified.

Reviewer #1: None

Reviewer #2: Yes

Reviewer #3: Yes

**********

PLOS authors have the option to publish the peer review history of their article (what does this mean?). If published, this will include your full peer review and any attached files.

If you choose “no”, your identity will remain anonymous but your review may still be made public.

**Do you want your identity to be public for this peer review?**  For information about this choice, including consent withdrawal, please see our Privacy Policy.

Reviewer #1: No

Reviewer #2: **Yes:**  Samuele Ramellini

Reviewer #3: No

[NOTE: If reviewer comments were submitted as an attachment file, they will be attached to this email and accessible via the submission site. Please log into your account, locate the manuscript record, and check for the action link "View Attachments". If this link does not appear, there are no attachment files.]

**Figure resubmission:**

**Reproducibility:**

?>

# Author comment: Author response to Decision Letter 2

**DOI:** 10.1371/journal.pcbi.1014875.r004

30 Sep 2026

Attachment Submitted filename: PCOMPBIOL_R2_response letter_v5.docx

# Editor report: Decision Letter 2

**DOI:** 10.1371/journal.pcbi.1014875.r005

**Authors:** Natalia Komarova

4 Oct 2026

Dear Dr van der Marel,

We are pleased to inform you that your manuscript 'Social manipulations trigger shifts in group-level dominance patterns' has been provisionally accepted for publication in PLOS Computational Biology.

Before your manuscript can be formally accepted you will need to complete some formatting changes, which you will receive in a follow up email. A member of our team will be in touch with a set of requests.

Please note that your manuscript will not be scheduled for publication until you have made the required changes, so a swift response is appreciated.

IMPORTANT: The editorial review process is now complete. PLOS will only permit corrections to spelling, formatting or significant scientific errors from this point onwards. Requests for major changes, or any which affect the scientific understanding of your work, will cause delays to the publication date of your manuscript.

Should you, your institution's press office or the journal office choose to press release your paper, you will automatically be opted out of early publication. We ask that you notify us now if you or your institution is planning to press release the article. All press must be co-ordinated with PLOS.

Thank you again for supporting Open Access publishing; we are looking forward to publishing your work in PLOS Computational Biology.

Best regards,

Natalia L. Komarova

Section Editor

PLOS Computational Biology

Natalia Komarova

Section Editor

PLOS Computational Biology

***********************************************************

# Editor report: Acceptance letter

**DOI:** 10.1371/journal.pcbi.1014875.r006

**Authors:** Natalia Komarova

PCOMPBIOL-D-26-00608R2

Social manipulations trigger shifts in group-level dominance patterns

Dear Dr van der Marel,

I am pleased to inform you that your manuscript has been formally accepted for publication in PLOS Computational Biology. Your manuscript is now with our production department and you will be notified of the publication date in due course.

The corresponding author will soon be receiving a typeset proof for review, to ensure errors have not been introduced during production. Please review the PDF proof of your manuscript carefully, as this is the last chance to correct any errors. Please note that major changes, or those which affect the scientific understanding of the work, will likely cause delays to the publication date of your manuscript.

Soon after your final files are uploaded, unless you have opted out, the early version of your manuscript will be published online. The date of the early version will be your article's publication date. The final article will be published to the same URL, and all versions of the paper will be accessible to readers.

For Research, Software, and Methods articles, you will receive an invoice from PLOS for your publication fee after your manuscript has reached the completed accept phase. If you receive an email requesting payment before acceptance or for any other service, this may be a phishing scheme. Learn how to identify phishing emails and protect your accounts at https://explore.plos.org/phishing.

Thank you again for supporting PLOS Computational Biology and open-access publishing. We are looking forward to publishing your work!

With kind regards,

Sharmila Kamatchi

PLOS Computational Biology | Carlyle House, Carlyle Road, Cambridge CB4 3DN | United Kingdom ploscompbiol@plos.org | Phone +44 (0) 1223-442824 | ploscompbiol.org | @PLOSCompBiol
